# Facile preparation of salivary extracellular vesicles for cancer proteomics

**DOI:** 10.1038/srep24669

**Published:** 2016-04-19

**Authors:** Yan Sun, Zhijun Xia, Zhi Shang, Kaibo Sun, Xiaomin Niu, Liqiang Qian, Liu-Yin Fan, Cheng-Xi Cao, Hua Xiao

**Affiliations:** 1 State Key Laboratory of Microbial Metabolism, Laboratory of Analytical Biochemistry and Bioseparation, School of Life Sciences and Biotechnology, Shanghai Jiao Tong University, Shanghai, 200240, China; 2 Department of Shanghai Lung Cancer Center, Shanghai Chest Hospital, Shanghai Jiao Tong University, Shanghai, 200030, China

## Abstract

Extracellular vesicles (EVs) are membrane surrounded structures released by cells, which have been increasingly recognized as mediators of intercellular communication. Recent reports indicate that EVs participate in important biological processes and could serve as potential source for cancer biomarkers. As an attractive EVs source with merit of non-invasiveness, human saliva is a unique medium for clinical diagnostics. Thus, we proposed a facile approach to prepare salivary extracellular vesicles (SEVs). Affinity chromatography column combined with filter system (ACCF) was developed to efficiently remove the high abundant proteins and viscous interferences of saliva. Protein profiling in the SEVs obtained by this strategy was compared with conventional centrifugation method, which demonstrated that about 70% more SEVs proteins could be revealed. To explore its utility for cancer proteomics, we analyzed the proteome of SEVs in lung cancer patients and normal controls. Shotgun proteomic analysis illustrated that 113 and 95 proteins have been identified in cancer group and control group, respectively. Among those 63 proteins that have been consistently discovered only in cancer group, 12 proteins are lung cancer related. Our results demonstrated that SEVs prepared through the developed strategy are valuable samples for proteomics and could serve as a promising liquid biopsy for cancer.

Extracellular vesicles (EVs) are defined as intact, submicron, phospholipid-rich vesicles ranging from 100 nm to 1000 nm in diameters, which shed from the surface of cells^1^. The functions of EVs are not completely understood yet. They are initially known as garbage cans whose job is to discard unwanted cellular substances^2^,^3^. However, recent research has revealed that these vesicles act also as important messengers for intercellular communication^4^. For instance, they could putatively attach or fuse with the target cell membrane, delivering surface proteins and perhaps cytoplasm to the recipient cell^5^,^6^. These properties are critical for signal transduction in the microenvironments, especially in disease pathogenesis and the tumor organotropic metastasis^7^,^8^,^9^. Study of gliomas tumor cells demonstrated that microvesicles from tumor cells could release to cellular surroundings and blood of tumor-bearing mice and contribute to horizontal propagation of oncogenes^10^. Therefore, it is of great interest to explore the proteome of EVs that originate from human body fluids, which might carry important biomarkers for the early detection of cancers^11^.

Human saliva is an attractive body fluid for molecular diagnostics, due to its unique composition and non-invasive sample collection. Owing to its enormous diagnostic potential, human saliva has been comprehensively explored for the detection of different oral diseases^12^,^13^ as well as systemic diseases^14^,^15^,^16^,^17^,^18^. Meanwhile, saliva has been recommended as a detection medium by the FDA for vulnerable populations, for instance children. Of note is that human saliva harbors plenty of extracellular vesicles (EVs), namely salivary extracellular vesicles (SEVs)^19^,^20^. SEVs studies demonstrated that tumor-secreted vesicles could enter the extracellular microenvironment and then affect and alter salivary gland *in vitro*^21^ and *in vivo*^22^, More specifically, tumor cell-specific mRNA and protein could be detected in microvesicles from saliva and blood. Unique biological information of tumor carried by EVs could also initiate the proliferation and metastasis of lung cancer^23^, which emphasized the association between distal tumor progression and the biomarker discovery in saliva through microvesicles.

However, one of the obstacles for SEVs preparation is the interference of high abundant amylase and other viscous proteins in saliva^24^. The overwhelming concentration of amylase in saliva could affect the identification and characterization of low abundant proteins as well as SEVs proteins, which are often biomarker candidates^25^. Meanwhile, amylase also interfere the extraction and separation of EVs by encasing and clinging globule with membrane structures and viscous proteins. Thus removal of amylase and other viscous proteins from saliva before SEVs’ extraction could benefit downstream proteomic analysis of SEVs and contribute to biomarker discovery for cancer^26^.

Lung cancer is one of the leading cancers for both genders worldwide and the most common causes of cancer related deaths^27^. The incidence of lung cancer has significantly increased in recent years, partially owing to the large smoking population and the declined air quality^28^. In addition, the five-year survival for lung cancer was lower than 10%. This dismal prognosis is mainly due to the fact that most patients were diagnosed at III or IV stage of disease. Therefore, early detection is the key for cure and the most effective way to reduce lung cancer deaths^29^. However, it’s technically challenging for medical imaging techniques and invasive biopsy to find early cancers, due to the limited resolution and specificity^30^. Molecular diagnostics is a promising and alternative approach for the early detection of cancers. Of note is that proteomic biomarkers have been discovered in human saliva for the detection of lung cancer^31^.

Since there is no standard operation procedure for pre-treatment of saliva samples as well as SEVs preparation, it is therefore necessary to establish a straightforward method as that for serum to remove high abundant amylase and viscous proteins. Starch has been previously used to specifically remove amylase from saliva, based on their strong affinity interactions^32^. Especially, it is easily available, economical, and feasible for practical applications. Keeping the availability and cost of starch in mind, hereby we intent to develop an affinity chromatography column combined with a filter system (ACCF) to trap amylase specifically, using starch as the stationary phase, and enrich EVs in saliva samples. In the present work, we prepared SEVs by ACCF and compared their protein profiling with that isolated by the conventional centrifugation method. The developed approach was further applied to harvest SEVs from healthy subjects and lung cancer patients, respectively. The proteome of both groups were compared through shotgun proteomics and further used for candidate biomarker discovery for the detection of lung cancer.

## Results

Our human saliva contains high abundant proteins along with many viscous proteins, which interfere in the preparation of SEVs. To prepare quality SEVs for clinical applications and also minimize the interference of salivary proteins, herein we developed an affinity chromatography coupled with filter system aiming at high quality SEVs separation, which can be further used for cancer proteomics.

### ACCF system development

**Figure 1:**
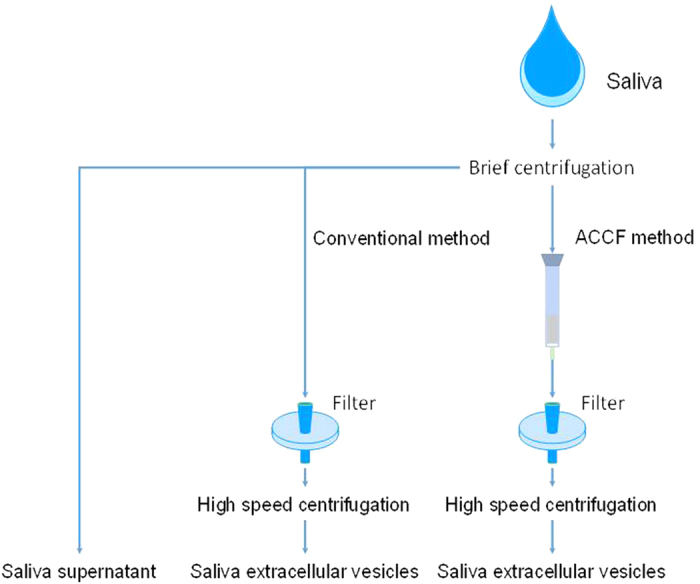
Schematic diagram for EVs isolation from human saliva.

The ACCF system consisted of two parts, the affinity chromatography column (ACC) and membrane filter (F), as shown in Fig. 1. The ACC part was a column prepared in a syringe by packing 0.5 g starch (from potato, Sigma, Shanghai, China) with 3 mL phosphate buffered saline (PBS) (Shanghai Bioscience Co. Ltd), which was adequate for 300 μL saliva sample preparation. The F part was a filter with 5 μm PVDF membrane (Millipore, Billerica, MA, USA).

**Figure 2:**
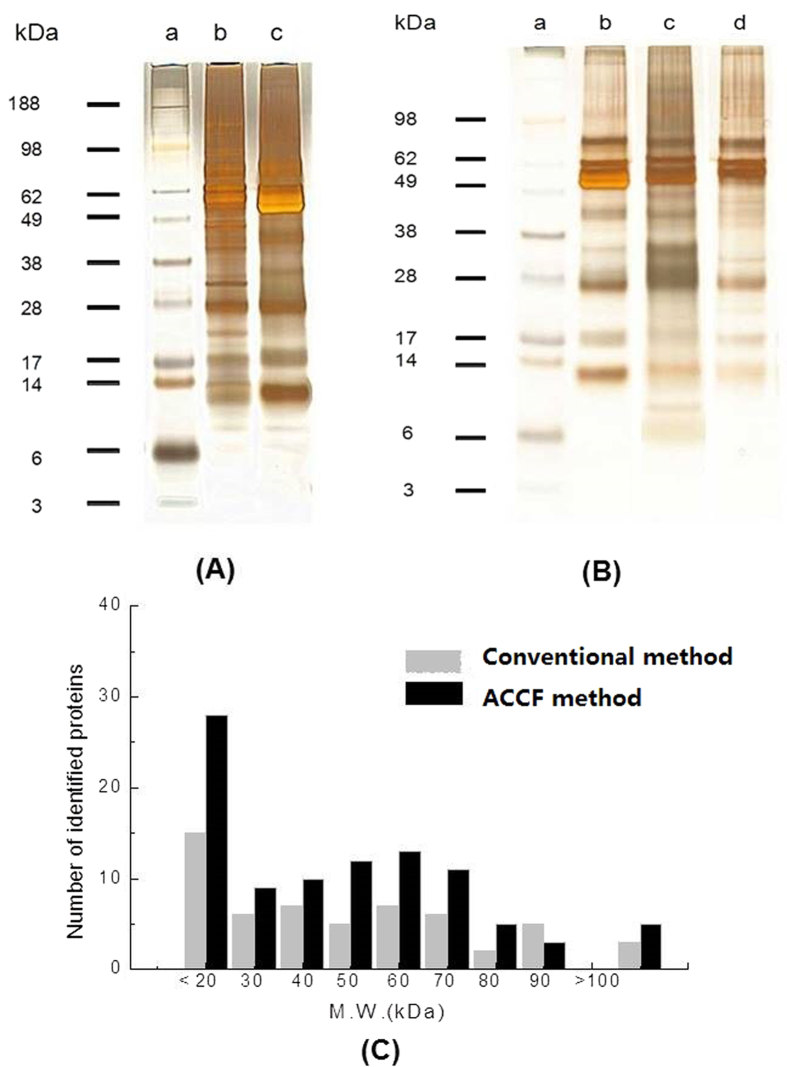
SDS-PAGE of salivary proteins and SEVs proteins. (**A**) 1D SDS-PAGE of salivary protein. (a) Protein ladder; (b) 1.5 μg of salivary proteins prepared by ACCF method; (c) 1.5 μg of primordial salivary proteins. (**B**) 1D SDS-PAGE of SEVs proteins. (a) Protein ladder; (b) 1.0 μg of salivary proteins; (c) 1.0 μg of EVs’ proteins prepared by ACCF method; (d) 1.0 μg of EVs’ proteins prepared by conventional centrifugation method. (**C**) Molecular weight distribution of SEVs proteins prepared by conventional centrifugation method and ACCF method.

In order to evaluate purification efficiency of the ACCF system, 1D SDS-PAGE was used to separate salivary and SEVs proteins, as shown in Fig. 2A. Comparing the protein bands obtained from SEVs isolated by ACCF (lane **b**) and saliva (lane **c**), notable difference appeared between 49 kDa and 62 kDa, because this range was mostly salivary amylase^33^. In lane (**c**), amylase was a wide and strong band while other protein bands were masked in the same region. In addition, lane (**b**) displayed more refined protein bands than lane (**c**), which suggests that high content amylase create interference in the separation and detection of other low abundant proteins.

In order to compare the ACCF system with the conventional centrifugation method, the extracted SEVs proteins from both ACCF and conventional centrifugation method were separated by SDS-PAGE (Fig. 2B). The SDS-PAGE analysis revealed that proteins isolated from SEVs by using ACCF (lane **c**) and conventional centrifugation method (lane **d**) exhibited significant difference in their profiling when compared to the whole saliva protein (lane **b**). It was observed that the SEVs’ proteins obtained from ACCF (lane **c**) have some unique compositions. When lane **d** was compared with lane **c**, it was observed that there are a large number of low molecule weight proteins (≤14 kDa). We quantitatively compared the salivary proteins before and after amylase removal and found that the salivary protein concentration decreased from 1.25 μg/μL to 0.62 μg/μL. This result confirmed that amylase is the most abundant protein in saliva, which could be trapped by ACCF system. The reproducibility of these two methods was evaluated by isolating SEVs from 300 μL saliva sample. As shown in Table 1, the RSD (n = 3) for ACCF method and conventional centrifugation method was 1.85% and 2.83%, respectively.

### Nanoparticle tracking analysis for the size distribution of SEVs

**Figure 3:**
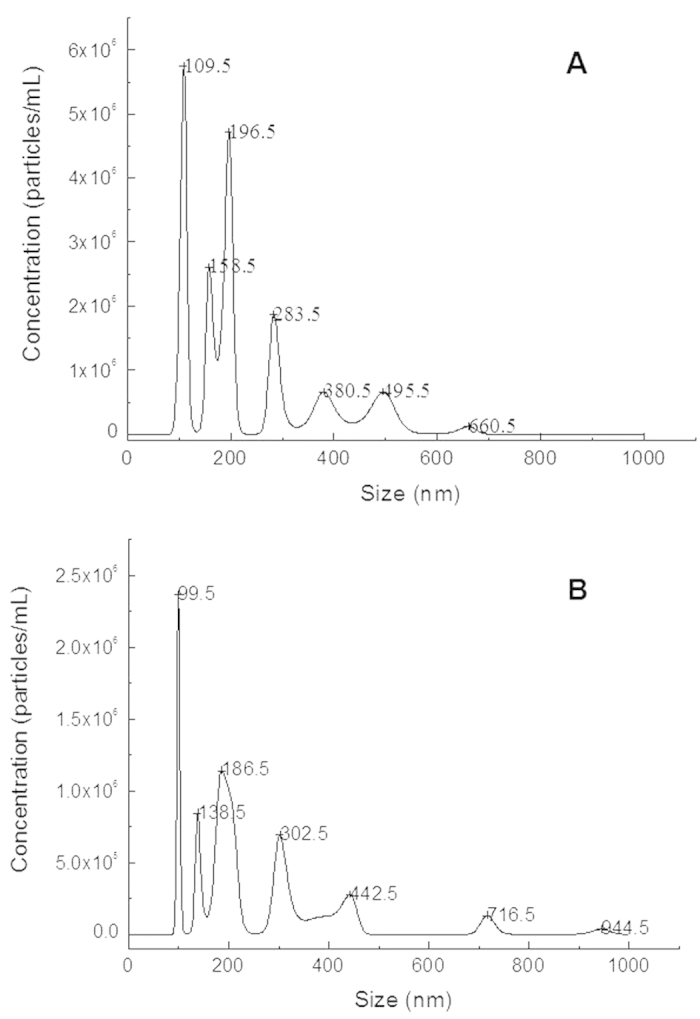
Particle size and distribution of the SEVs obtained by primordial saliva (**A**) and purified saliva sample using ACCF system (**B**).

Nanoparticle tracking analysis was used to evaluate the size distribution of SEVs. The results are shown in Fig. 3. The SEVs size in primordial saliva and purified saliva sample using ACCF system was both smaller than 1,000 nm. However, for SEVs prepared from conventional centrifugation method, the particle size ranged from 109 nm to 660 nm, while for SEVs prepared through ACCF method, the diameter ranged from 99 nm to 944 nm.

### Proteomics analysis of SEVs

**Figure 4:**
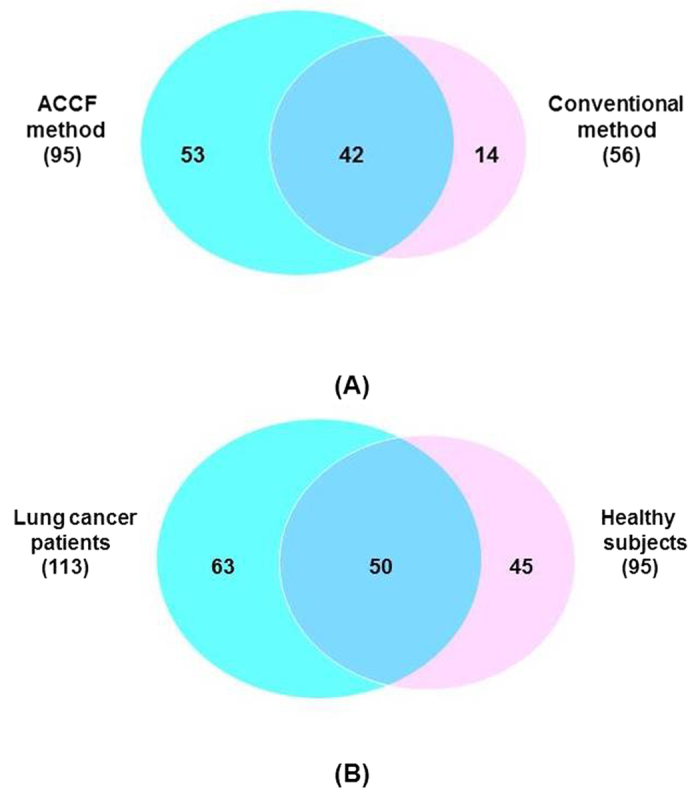
Venn diagram of identified proteins. (**A**) Overlap of SEVs proteins prepared through ACCF method and conventional method; (**B**) Overlap of SEVs proteins extracted from the saliva of lung cancer patients and healthy subjects.

LC-MS/MS based shotgun proteomic approach was applied for the proteome analysis of SEVs. All experiments (both conventional centrifugation method and ACCF method) were carried out in triplicate. For each SEVs sample obtained by ACCF method, we identified 128, 138 and 107 proteins, respectively. For each SEVs sample prepared by conventional centrifugation method, we discovered 87, 76 and 72 proteins, respectively. To extract the most reliable data, we used the overlapped results of 3 experiments. Finally, we consistently obtained 95 SEVs proteins for ACCF method and 56 SEVs proteins for conventional centrifugation method (Fig. 4). The number of identified SEVs proteins was increased more than 70% through using ACCF method (Fig. 4A). Further analysis revealed that 42 proteins were shared by both methods, which include SEVs marker protein Integrin beta-2^34^. The ACCF method was able to discover 53 unique proteins from SEVs, while 14 proteins were unique to conventional centrifugation method. It could be observed from Fig. 2C that the use of ACCF approach for the removal of amylase led to find more number of low molecule weight proteins (<20 kDa) than conventional centrifugation method. These results were consistent with the SDS-PAGE analysis of both methods (Fig. 2B).

**Figure 5:**
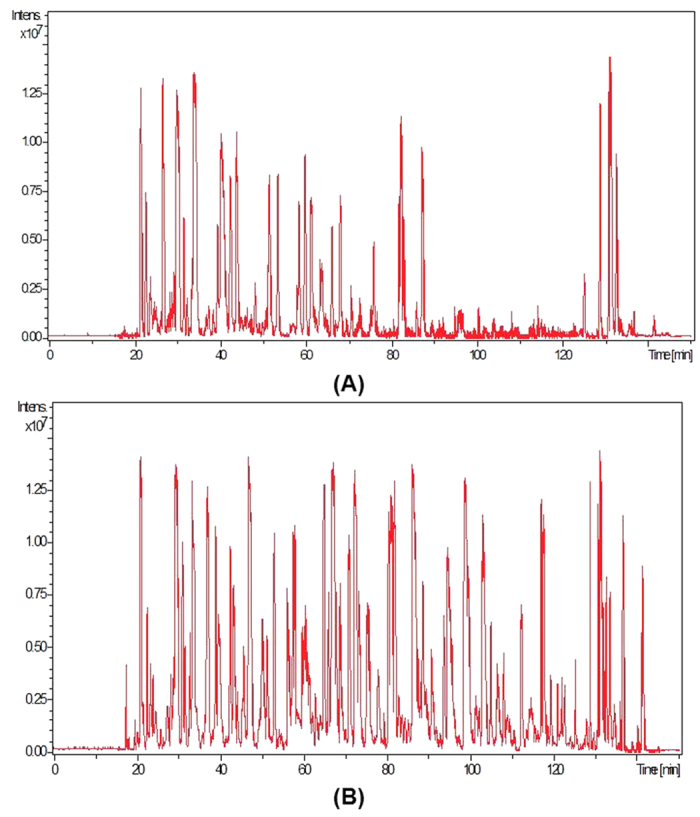
Total ion chromatogram of SEVs’ peptides from primordial saliva (**A**) and ACCF purified saliva (**B**).

To further demonstrate the interference of amylase on SEVs isolation and their protein identification, the total ion chromatograms of SEVs’ peptides prepared through ACCF method and the conventional one were compared, as shown in Fig. 5. The SEVs sample obtained after removal of amylase eluted more peaks between retention time of 90 mins and 130 mins (Fig. 5B), which partially contributed to the identification of 53 unique proteins for SEVs.

Detailed information of identified SEVs proteins through conventional centrifugation method and ACCF method is shown in Tables S1 and S2, respectively. To uncover the molecular events underlying these discrepant proteins profiles, we studied the Gene Ontology information of these specific proteins, which include biological process, cellular component, molecular function, pathway, and protein class analysis by using PANTHER software (Fig. S1). The Gene Ontology pathway information of SEVs proteins obtained by ACCF approach and conventional centrifugation method was compared. We found SEVs proteins discovered through conventional centrifugation method revealed only 6 common pathways which are necessary for common cell growth and immune cell activation. Whereas, SEVs proteins obtained by ACCF method involved in 31 pathways, indicating many dynamic disease processes.

### ACCF method for proteomic analysis of cancer SEVs

We further applied the ACCF system to isolate SEVs from the saliva of 3 lung cancer patients. SEVs proteins were extracted and analyzed by LC-MS/MS in parallel and technically repeated. We identified 138, 146, and 129 proteins, respectively, for the lung cancer SEVs sample prepared by ACCF method. We also used the overlapped data and 113 proteins (Table S3) were consistently discovered from the SEVs prepared from the saliva of cancer patients. The Gene Ontology data of these 113 proteins is shown in Fig. S2. When proteins were further compared with healthy subjects, 50 proteins were commonly shared by the two methods, 45 proteins presented uniquely in healthy subjects’ saliva and 63 proteins only appeared in lung cancer patients’ SEVs (Fig. 4B). The differences of SEVs’ protein between healthy subjects and lung cancer patients suggested that SEVs carried versatile biological information, which might serve as a biomarker source for lung cancer.

**Figure 6:**
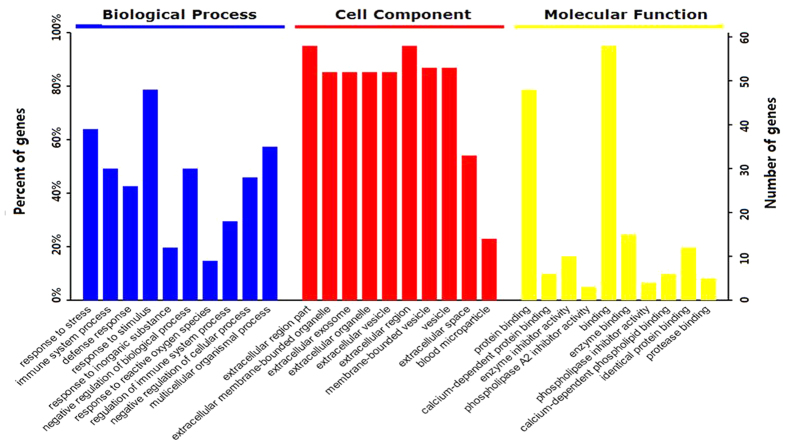
Gene Ontology analysis of candidate SEVs’ protein biomarkers for lung cancer.

To discover potential biomarkers, we further analyzed these 63 proteins that were unique to lung cancer (Table 2). The Gene Ontology information of these 63 proteins showed that about 80% were involved in response to stimulus and 60% were related to stress response and multicellular organismal process (Fig. 6). The Gene Ontology information further revealed the extracellular nature of these proteins, they were mainly present in region part, membrane-bounded organelle, vesicle or exosome and blood microparticle. Molecular function results of these proteins showed that 90% gene referred to binding, which includes protein binding, enzyme binding, identical protein binding, and protease binding.

**Figure 7:**
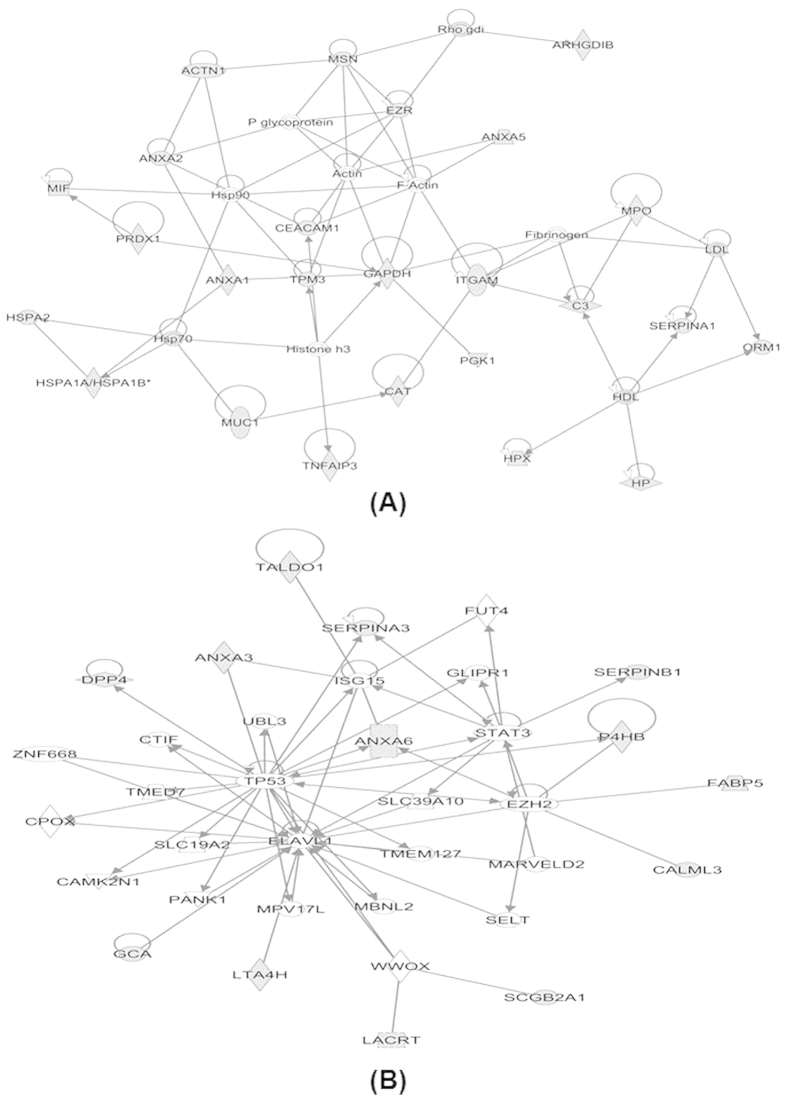
IPA analysis of the candidate SEVs’ protein biomarkers for lung cancer. Number of lung cancer specific proteins involve in cancer related network (**A**) and cellular movement related network (**B**). Gray symbols are SEVs proteins identified in this study.

Ingenuity Pathway Analysis (IPA, Redwood City, CA, USA) analysis of these candidate SEVs’ protein biomarkers for lung cancer revealed that 25 of them (about 40%) involved in cancer network (Fig. 7A) and 13 of them (about 20%) were related with cellular movement (Fig. 7B, Table S4). Further, the literature survey showed that out of these 40% proteins, 12 proteins are lung cancer related biomarkers, which includes Annexin family members (Annexin A1, A2, A3, A5, A6, A11), Nitrogen permease regulator 2-like protein (NPRL2), Carcinoembryonic antigen-related cell adhesion molecule 1 (CEACAM1), Mucin 1 (MUC1), Prominin-1 (PROM1), Histone H4 (HIST1H4A), and Tumor necrosis factor alpha-induced protein 3 (TNFAIP3).

## Discussion and Conclusion

EVs have emerged as a potential biomarker source for molecular diagnostics. However, due to their small size and low abundance in body fluids, it is very challenging to efficiently separate and prepare EVs. In particular, human saliva contains high abundant proteins and viscous components that could disturb the extraction of EVs by encapsulating and overshadowing the EVs. In this case, we assumed that a portion of EVs might be attached to or gathered on proteins such as amylase and mucins hence decreasing the yield of EVs extraction. And protein aggregations with similar size of EVs may interfere in the accuracy and the quantity of proteomic analysis. To overcome these SEVs extraction issues, we developed ACCF technique in which on one hand we can remove high abundance amylase to reduce the protein interference. On the other hand, the system can filtrate out aggregated protein to release more types of EVs.

In this study, we paid much attention to the isolation of EVs and EVs proteins from saliva. We chose starch as an affinity stationary phase to specifically remove salivary amylase. After removal of amylase from saliva, we further used the processed saliva sample to prepare EVs. We observed that SEVs isolated by ACCF approach have broader diameter range (Fig. 3), which indicated that different types of SEVs were isolated. Therefore, our approach could further increase the quantity of SEVs proteins and boost their comprehensive proteomic analysis (Figs 4 and 5). Meanwhile, according to the SDS-PAGE results shown in Fig. 2, we found that low abundant proteins in saliva samples could be well resolved after amylase removal. In addition, more protein bands could be observed for SEVs proteins prepared by ACCF method than that of conventional method, especially many low molecular weight proteins as discovered by LC-MS/MS.

We further applied the developed method to prepare SEVs from lung cancer patient’ saliva as well as healthy subjects’ saliva and compared their protein profiling. We found 63 proteins that were unique to lung cancer patients (Table 2). After Gene Ontology analysis and extensive literature search we found that 12 of them were lung cancer related biomarkers, including 6 ANAX protein family members ANXA1, ANXA2, ANXA3, ANXA5, ANXA6, ANXA11. These ANAX proteins are associated with cell migration and vesicles fusion^35^,^36^. Another lung cancer related protein we found in cancer patients is NPRL2, which is a novel tumor suppressor gene and associated with cell growth and enhances sensitivity to various anticancer drugs^37^. CEACAM-1 and MUC1 were also presented in patients’ SEVs which mainly possess protein homodimerization activity and has been implicated in non-small-cell lung cancer development and progression^38^ and cancer cell signaling by promoting the synthesis and secretion of vascular endothelial growth factor (VEGF) through the AKT signaling pathway^39^.

Other lung cancer related proteins are PROM1, HIST1H4A and TNFAIP3. Exosome carried PROM1 has been one of the lipid raft-associated component that contributed to signal transduction and mediating intercellular communication^40^,^41^. HIST1H4A plays an important role in inducing cell death of tumor cells^42^. TNFAIP3 is an ubiquitin-editing enzyme, which is linked to a radioresistant phenotype of non-small cell lung cancer^43^.

Further EVs are known as signal transduction messengers. It has been proposed that EVs could assist tumor metastasis and diffusion after they were secreted to circulating system. Therefore, it is believed that EVs released from lung cancer tumor could carry tumor cell-specific proteins and enter saliva from blood, which has been verified in a xenografted mouse model of human lung cancer. In our study, lung cancer related proteins were identified in SEVs, which confirmed that these EVs in saliva might originate from lung cancer tumor. Therefore it is very promising to identify biomarkers from SEVs for the detection of lung cancer as well as other systemic diseases. Although further validation in a large sample set is required, the finding of candidate biomarkers in SEVs would provide a clue to the biological study of lung cancer and assist in future disease progression research. Our developed method for SEVs preparation will further expand the application of salivary diagnostics.

## Materials and Methods

### Saliva collection

Saliva samples were collected according to approved protocols (IRB#M15017) by Institutional Review Board (IRB) of Shanghai Jiao Tong University and all subjects provided written informed consents. The methods were carried out in accordance with the approved guidelines. All experimental protocols were approved by Bio-X Ethics Committee of Shanghai Jiao Tong University. Six healthy subjects and three lung cancer patients were recruited for this study. None of the healthy subjects had any history of malignancy, immunodeficiency, autoimmune disorders, hepatitis, and/or HIV infection. The whole saliva sample collection was performed as previously described. Briefly, saliva samples were pooled and kept on ice during the sample collection. Whole saliva sample was centrifuged at 2600 × g for 30 min at 4 °C to remove cells, bacteria, debris and food remnants, then protease inhibitor cocktail (Roche complete tablet, Roche Diagnostics GmbH, Roche Applied Science, Mannheim, Germany) was added to saliva supernatant to prevent protein degradation. Finally, the saliva sample was diluted with PBS at the ratio of 1:1 and ready for SEVs preparation.

### SEVs preparation

To prepare SEVs by using conventional centrifugation method, the diluted saliva supernatant was filtered with 5 μm PVDF membrane and then directly centrifuged to collect EVs. In our developed method, diluted saliva supernatant was loaded into ACCF system to remove amylase. Then the clear filtrate was centrifuged at 20,000 × g for 1 h at 4 °C to collect the EVs. The pellets were washed with PBS twice and then centrifuged again at 20,000 × g for another 1 h at 4 °C to harvest the final SEVs (Fig. 1).

### SEVs protein extraction

Protein extraction was the same as previously described. Briefly, the obtained SEVs were resuspended in 100 μl PBS afterwards 2 μL Triton X-100 and 5 μL protease inhibitor cocktails were added. Then the sample was placed on ice for 30 min to disrupt the membranes and centrifuged at 20,000 × g 1 h at 4 °C to remove the sediment. The soluble fraction was collected and precipitated with 10 times pre-chilled ethanol at −20 °C for 10 h. SEVs protein was further isolated by centrifugation at 15,000 × g for 30 min. Protein concentration was measured using the BCA method (BCA assay kit, Peirce, Rockford, USA).

### 1D SDS-PAGE and in-solution digestion

SEVs proteins were loaded into a 10% Bis-Tris Mini Gel (Life Technologies, Shanghai, China) and were run at 100 V for 60 min in MES SDS running buffer. Pre-stained protein standard (Life Technologies, Shanghai, China) was used to track protein migration. The resulting gels were stained with Fast Sliver Stain Kit (Beyotime, Beijing, China). In-solution tryptic digestion was carried out overnight at 37 °C using trypsin (Promega, Madison, WI, USA) in 50 μL 50 mM NH_4_HCO_3_.

### Nanoparticle tracking analysis

Nanoparticle tracking analysis (NTA, London, United Kingdom) offers the ability to directly visualize the size and count nanoparticles in liquid suspension. SEVs’ size was measured by the nanoparticle tracking system NanoSight (NTA: LM10, London, United Kingdom) by loading SEVs prepared from 1 mL saliva.

### Nano LC-MS/MS and database search

For shotgun proteomics, 10 μg proteins were taken from each cancer sample and control sample and processed in parallel. For protein identification, 1 μg protein digests from each sample were analyzed using an LC system (Nano Pump, Ultimate 3000, Dionex, Thermofisher) coupled with an ESI-Q-TOF mass spectrometer (Maxis Impact UHR Q-TOF, Impact, Bruker Daltonik, Germany). Briefly, each peptide sample was re-dissolved in 2% acetonitrile with 0.1% formic acid, and then loaded onto a peptide trap column (100 μm × 2 cm, 5 μm, Dionex, Thermofisher). Then trapped peptides were eluted to a C18 capillary column (75 μm × 15 cm, 3 μm, Dionex, Thermofisher). The peptides were eluted for 90 min with a gradient of 2–80% v/v of acetonitrile containing 0.1% v/v formic acid at flow rate of 400 nL/min. MS was performed in a positive mode using a repetitively full MS scan, followed by collision-induced dissociation of the five most dominant ions selected from the initial MS scan. The analysis of each sample was technically repeated in triplicates. The mass spectrometry proteomics data have been deposited to the ProteomeXchange Consortium via the PRIDE partner repository (http://www.ebi.ac.uk/pride/) with the dataset identifier PXD003269 and 10.6019/PXD003269.

Combined MS and MS/MS spectra were submitted for database search using MASCOT software (version 2.0) to identify proteins from the Human Swissprot database (548208 sequences). The peak list was directly generated from raw data using centroid algorithm with peak width set as 0.1 m/z and intensity above 100. No peak smooth or filter process was applied. The parameters for searching were enzyme of trypsin, 2 missed cleavage, fixed modifications of carbamidomethyl (C), and variable modifications of oxidation (M). A mass tolerance of 20 ppm was used for MS precursors and 0.05 Da for fragment ions. Peptide charges of +2, +3 and +4 were selected. The criteria of two peptides and C.I.% > 95 were used for protein identification, which allowed a 99% confidence level of protein identification with less than 1% false discovery rate.

## Additional Information

**How to cite this article**: Sun, Y. *et al.* Facile preparation of salivary extracellular vesicles for cancer proteomics. *Sci. Rep.* **6**, 24669; doi: 10.1038/srep24669 (2016).

## Supplementary Material

Supplementary Information

## Figures and Tables

**Table 1:** Reproducibility of the SEVs isolation.

Methods	No.	SEVs protein (μg)	Average	SD	RSD (%)
ACCF method	1	3.876	3.940	0.073	1.85
	2	3.926			
	3	4.020			
Conventional centrifugation method	1	3.324	3.315	0.094	2.83
	2	3.405			
	3	3.216			

SEVs were separated from 300 μL saliva sample by ACCF and conventional centrifugation method, respectively.

**Table 2:** List of candidate SEVs’ protein biomarkers for lung cancer.

No.	Accession#	Protein name	Gene symbol	M.W. (kDa)	#Unique peptide	Mascot score
**1**	**Q8WTW4**	**Nitrogen permease regulator 2-like protein**	**NPRL2**	**43.6**	**2**	**315**
2	P01009	Alpha-1-antitrypsin	A1AT	46.7	6	519
3	P01023	Alpha-2-macroglobulin	A2M	163.3	3	270
4	P01011	Alpha-1-antichymotrypsin	AACT	47.6	4	82
5	P12814	Alpha-actinin-1	ACTN1	103	2	269
6	P43353	Aldehyde dehydrogenase family 3 member B1	AL3B1	51.8	2	75
**7**	**P50995**	**Annexin A11**	**ANXA11**	**54.3**	**3**	**141**
**8**	**P04083**	**Annexin A1**	**ANXA1**	**38.7**	**5**	**435**
**9**	**P07355**	**Annexin A2**	**ANXA2**	**38.6**	**5**	**575**
**10**	**P12429**	**Annexin A3**	**ANXA3**	**36.3**	**2**	**460**
**11**	**P08758**	**Annexin A5**	**ANXA5**	**35.9**	**2**	**295**
**12**	**P08133**	**Annexin A6**	**ANXA6**	**75.8**	**4**	**159**
13	P84077	ADP-ribosylation factor 1	ARF1	20.6	2	264
14	P52566	Rho GDP-dissociation inhibitor 2	ARHGDIB	22.9	2	305
15	P01024	Complement C3	C3	187.1	5	450
16	Q5SNV9	Uncharacterized protein C1orf167	CA167	162.4	3	125
17	P27482	Calmodulin-like protein 3	CALL3	16.8	2	93
18	P04040	Catalase	CATA	59.7	3	92
19	P07339	Cathepsin D	CATD	44.5	2	90
**20**	**P13688**	**Carcinoembryonic antigen-related cell adhesion molecule 1**	**CEACAM1**	**57.5**	**7**	**671**
21	P01034	Cystatin-C	CYTC	15.7	2	126
22	P27487	Dipeptidyl peptidase 4	DPP4	88.2	3	85
23	P15311	Ezrin	EZRI	69.4	2	43
24	Q01469	Fatty acid-binding protein, epidermal	FABP5	15.1	3	502
25	P15328	Folate receptor alpha	FOLR1	29.8	4	81
26	P04406	Glyceraldehyde-3-phosphate dehydrogenase	G3P	36	2	250
27	P28676	Grancalcin	GRAN	24	2	71
28	Q58FF8	Putative heat shock protein HSP 90-beta 2	H90B2	44.4	2	33
29	Q96A08	Histone H2B type 1-A	HIST1H2BA	14.1	11	1002
**30**	**P62805**	**Histone H4**	**HIST1H4A**	**11.3**	**4**	**402**
31	P00738	Haptoglobin	HPT	45.2	3	75
32	P02790	Hemopexin	HPX	51.6	6	560
33	Q58FG1	Putative heat shock protein HSP 90-alpha A4	HS904	47.7	2	36
34	P54652	Heat shock-related 70 kDa protein 2	HSP72	70	2	147
35	P0DMV8	Heat shock 70 kDa protein 1A	HSPA1A	70	4	402
36	P0DMV9	Heat shock 70 kDa protein 1B	HSPA1B	70	4	365
37	P48741	Putative heat shock 70 kDa protein 7	HSPA7	40.2	3	43
38	P01781	Ig heavy chain V-III region GAL	HV320	12.7	3	245
39	P30740	Leukocyte elastase inhibitor	ILEU	42.7	2	194
40	P11215	Integrin alpha-M	ITAM	127.1	5	301
41	P01593	Ig kappa chain V-I region AG	KV101	12	2	160
42	Q9GZZ8	Extracellular glycoprotein lacritin	LACRT	14.2	7	614
43	P80188	Neutrophil gelatinase-as	LCN2	22.5	3	397
44	P00338	L-lactate dehydrogenase A chain	LDHA	36.6	4	115
45	P09960	Leukotriene A-4 hydrolase	LKHA4	69.2	2	227
46	P14174	Macrophage migration inhibitory factor	MIF	12.4	6	86
47	Q13421	Mesothelin	MSLN	68.9	8	805
48	P26038	Moesin	MSN	67.8	9	713
**49**	**P15941**	**Mucin-1**	**MUC1**	**122.1**	**4**	**390**
50	P02763	Alpha-1-acid glycoprotein 1	ORM1	23.5	4	410
51	P07237	Protein disulfide-isomerase	PDIA1	57.1	2	121
52	P05164	Myeloperoxidase	PERM	83.8	2	97
53	P00558	Phosphoglycerate kinase 1	PGK1	44.6	4	217
54	Q06830	Peroxiredoxin-1	PRDX1	22.1	4	104
**55**	**O43490**	**Prominin-1**	**PROM1**	**97.2**	**8**	**705**
56	Q6MZM9	Proline-rich protein 27	PRR27	22.7	2	46
57	O75556	Mammaglobin-B	SCGB2A1	10.8	4	330
58	Q96QR1	Secretoglobin family 3A member 1	SCGB3A1	10.1	5	528
59	Q687×5	Metalloreductase STEAP4	STEA4	51.9	2	80
60	P37837	Transaldolase	TALDO	37.5	2	58
61	P20061	Transcobalamin-1	TCO1	20.6	4	219
**62**	**P21580**	**Tumor necrosis factor alpha-induced protein 3**	**TNFAIP3**	**89.6**	**2**	**335**
63	P06753	Tropomyosin alpha-3 chain	TPM3	32.9	3	285
