# Supplementary material for: Facile preparation of salivary extracellular vesicles for cancer proteomics
Source: Sci Rep. 2016 Apr 19;6:24669. doi: 10.1038/srep24669 (PMC4835767; doi:10.1038/srep24669)
Supplement: Supplementary Information [file srep24669-s1.doc]

Supplementary Materials for

**Facile preparation of salivary extracellular vesicles for cancer proteomics**

Yan Sun ^a^, Zhijun Xia ^a^, Zhi Shang ^a^, Kaibo Sun ^a^, Xiaomin Niu ^b^, Liqiang Qian ^b^, Liu-Yin Fan ^a^, Cheng-Xi Cao ^a,*^, and Hua Xiao ^a,*^

**Figure S1.** Gene Ontology analysis of SEVs’ proteins discovered using ACCF method and conventional method. (A) Biological process; (B) cellular component; (C) molecular function; (D) pathway; (E) protein class.

| ACCF method | General method |
| --- | --- |
| **（A1）** | **（A2）** |
| 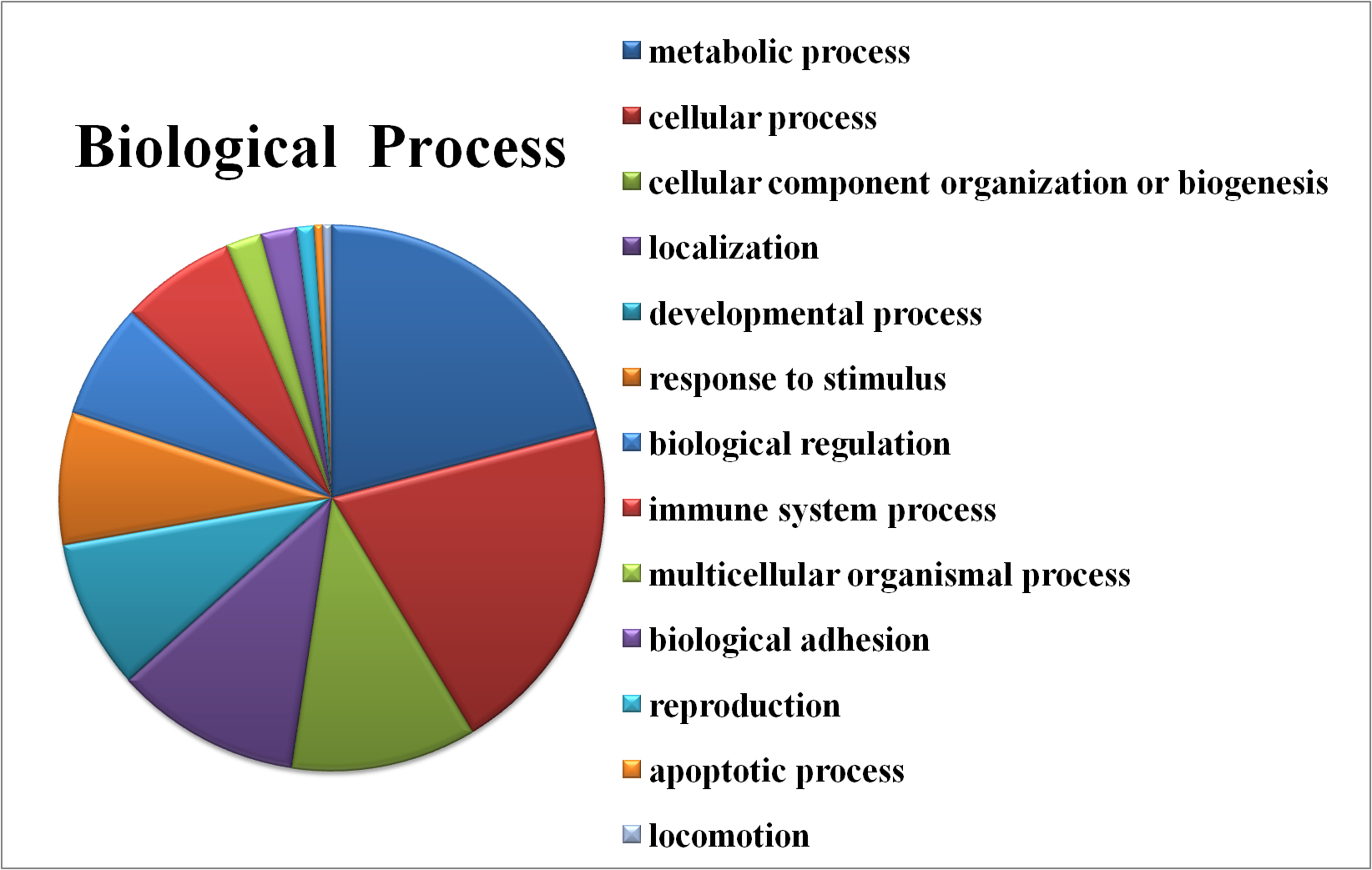 | 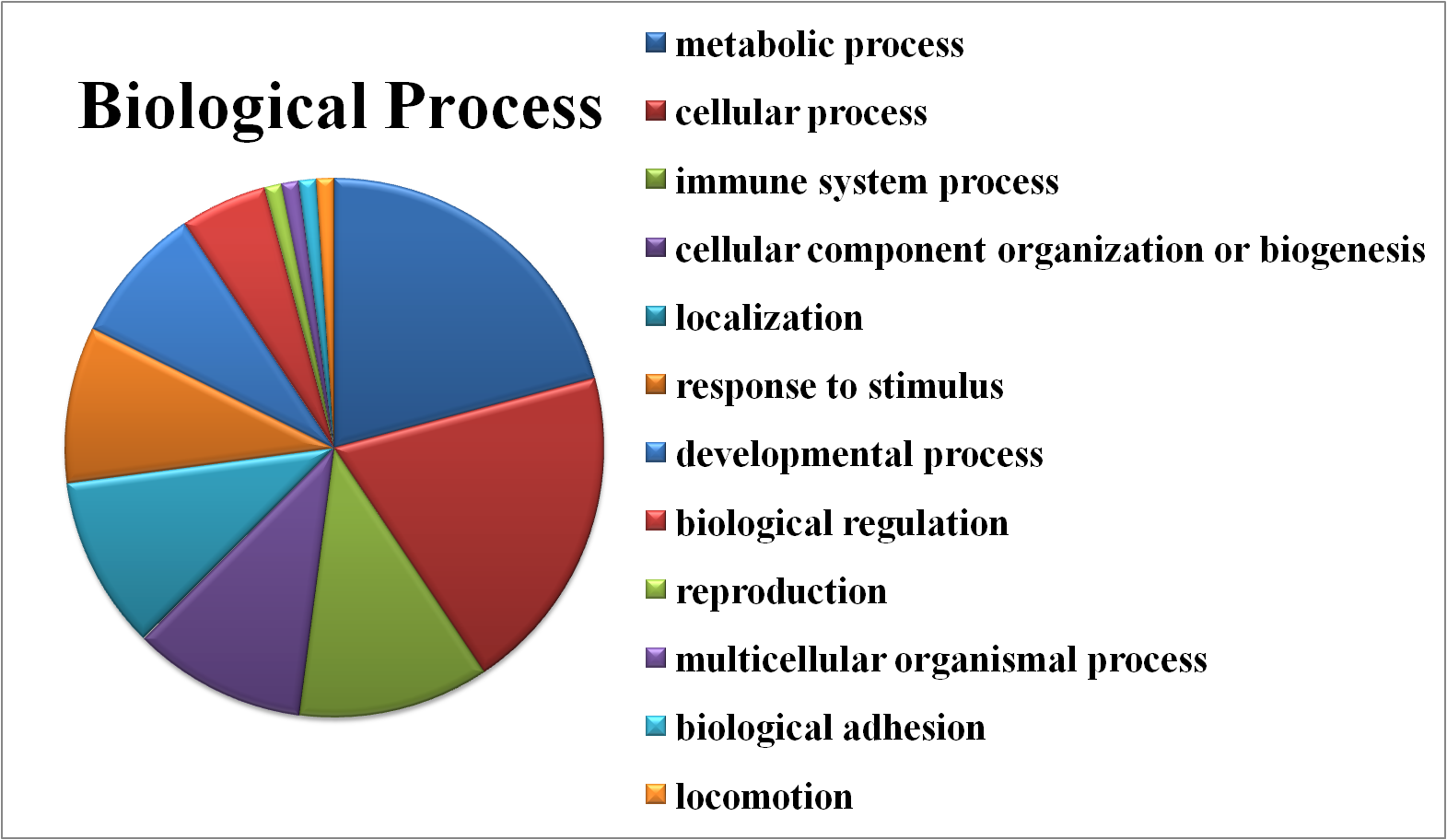 |
| **（B1）** | **（B2）** |
| 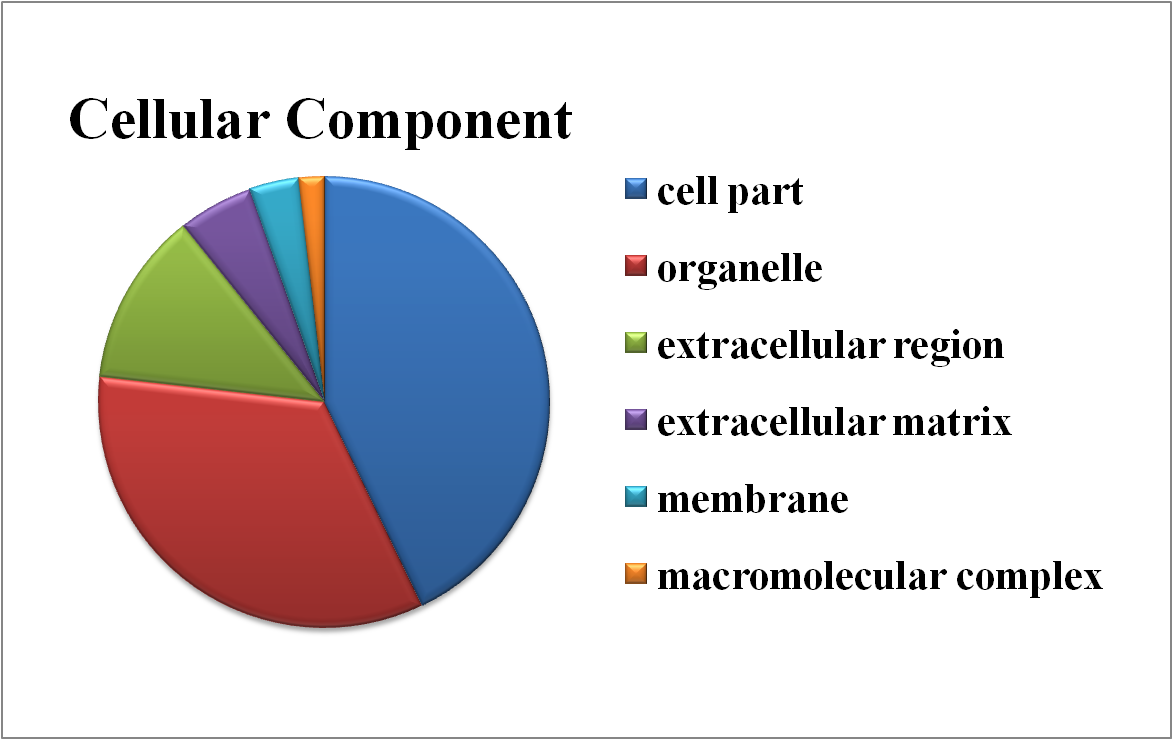 | 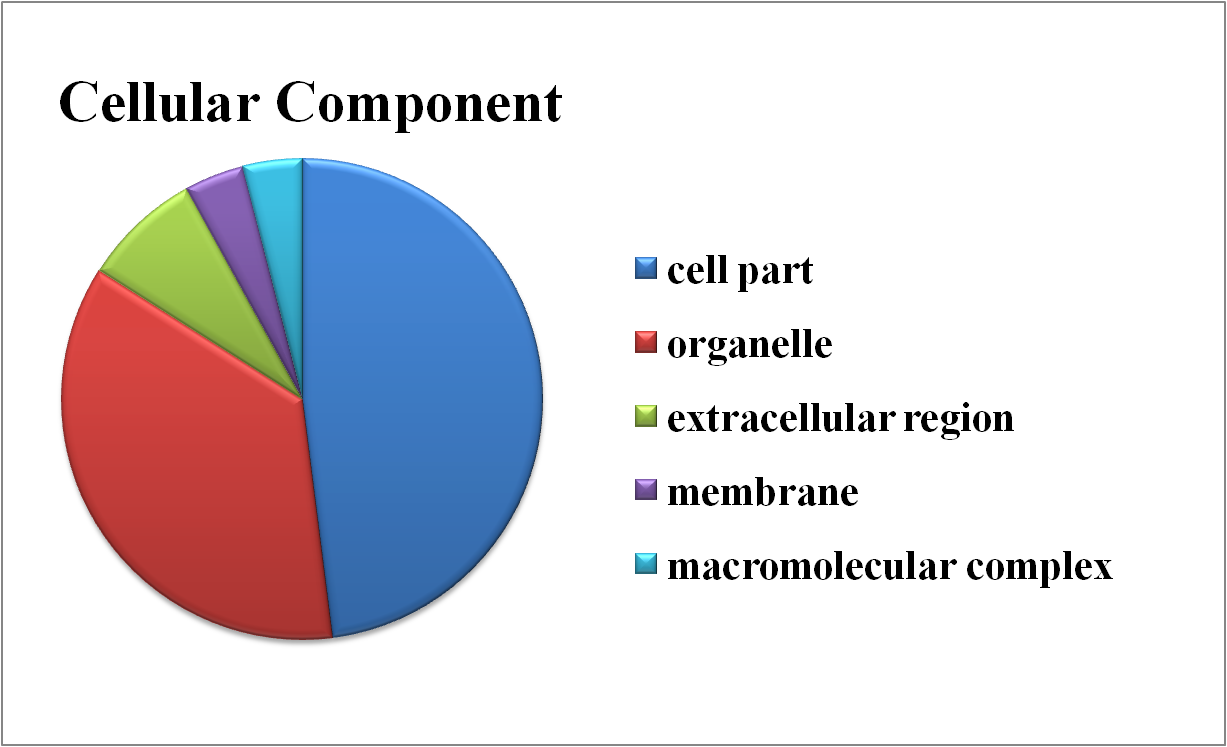 |
| **（C1）** | **（C2）** |
| 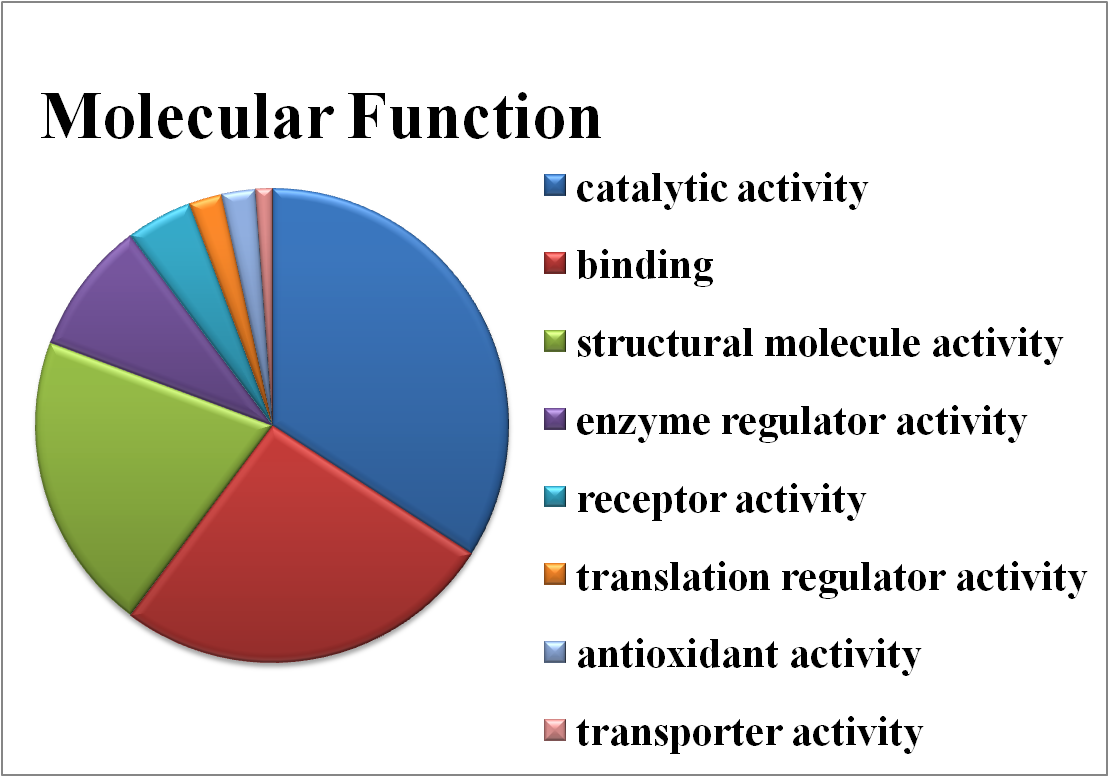 | 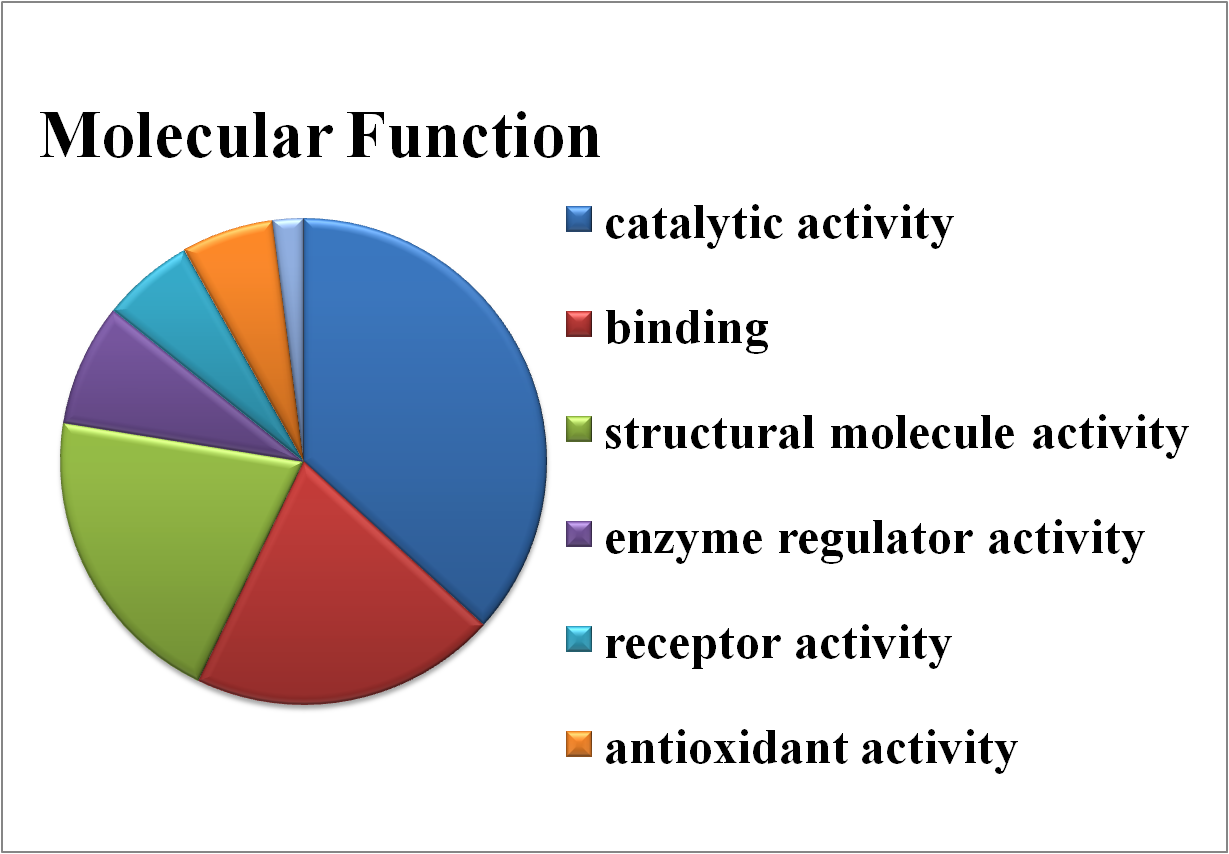 |
| **（D1）** | **（D2）** |
| 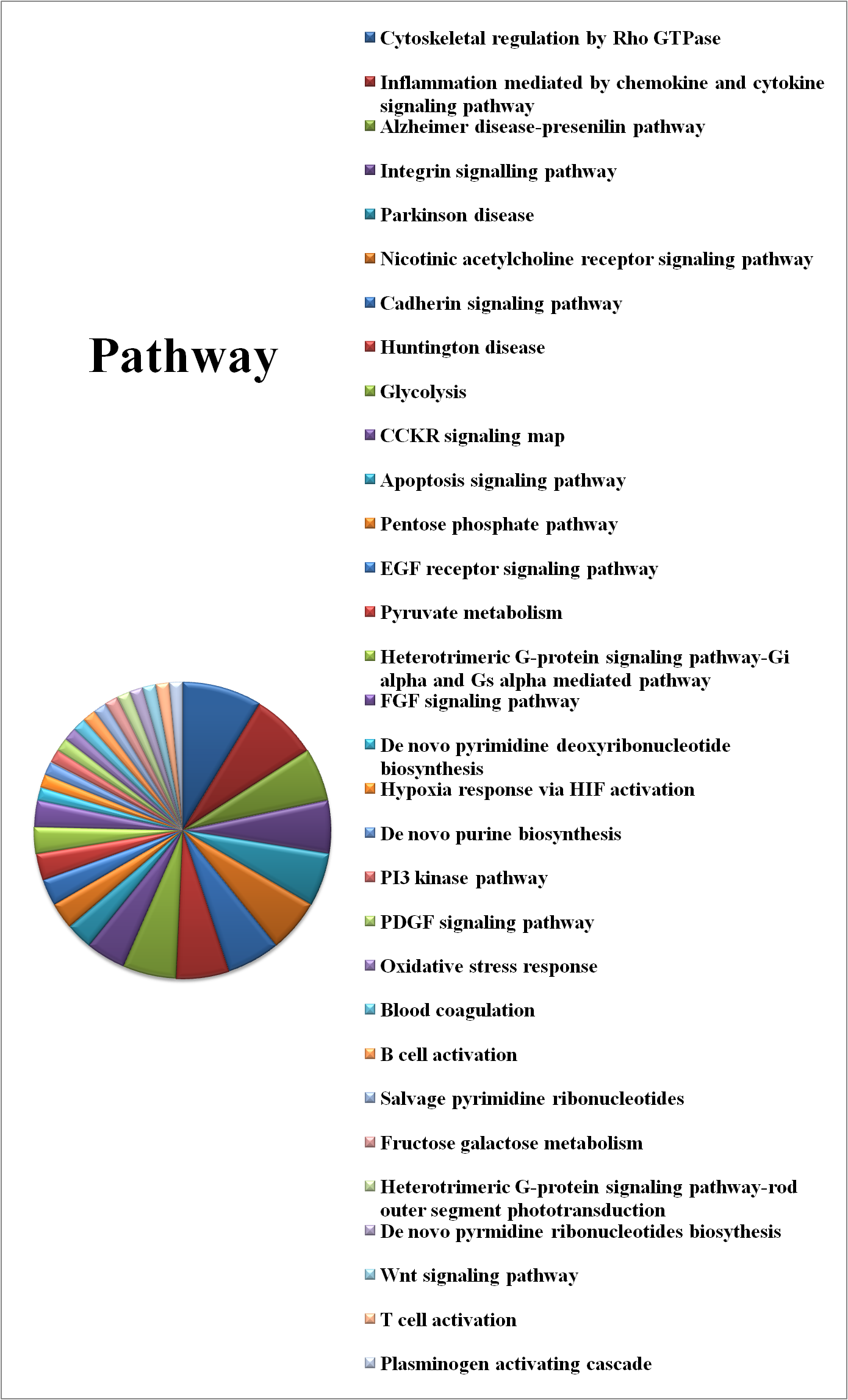 | 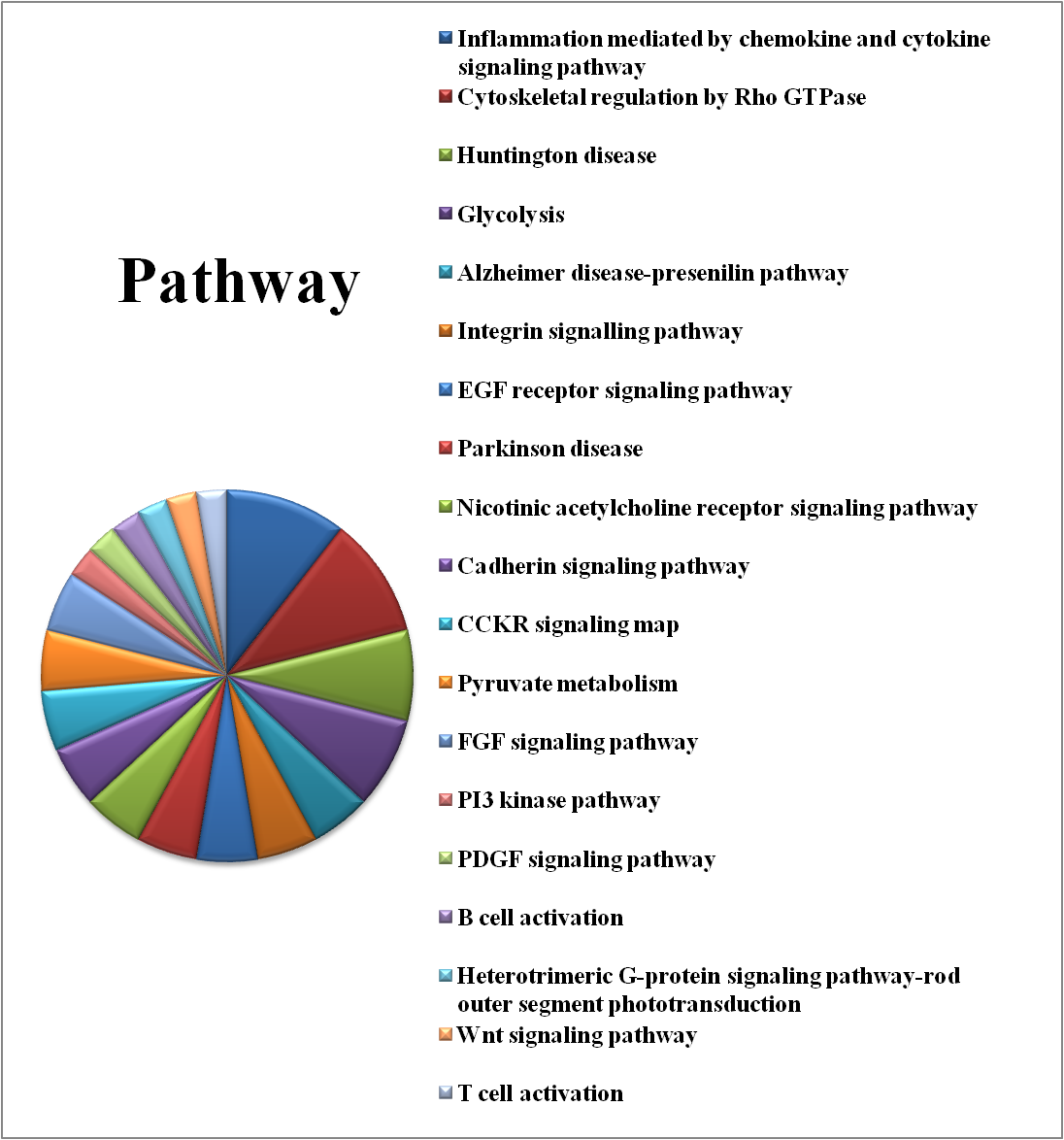 |
| **（E1）** | **（E2）** |
| 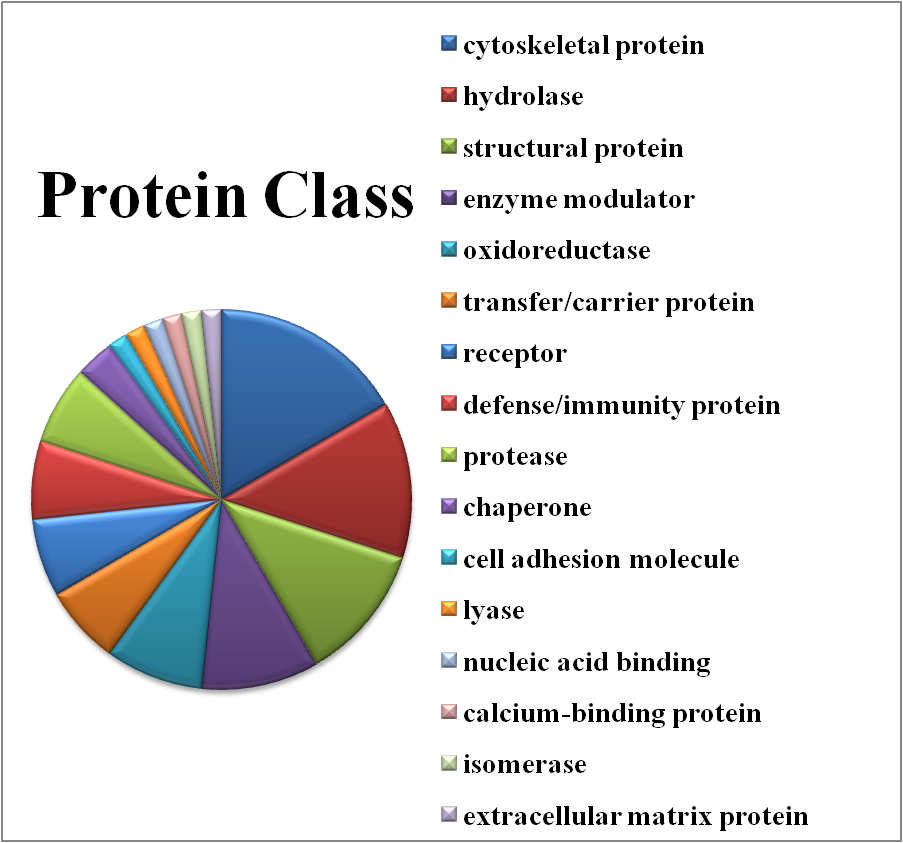 | 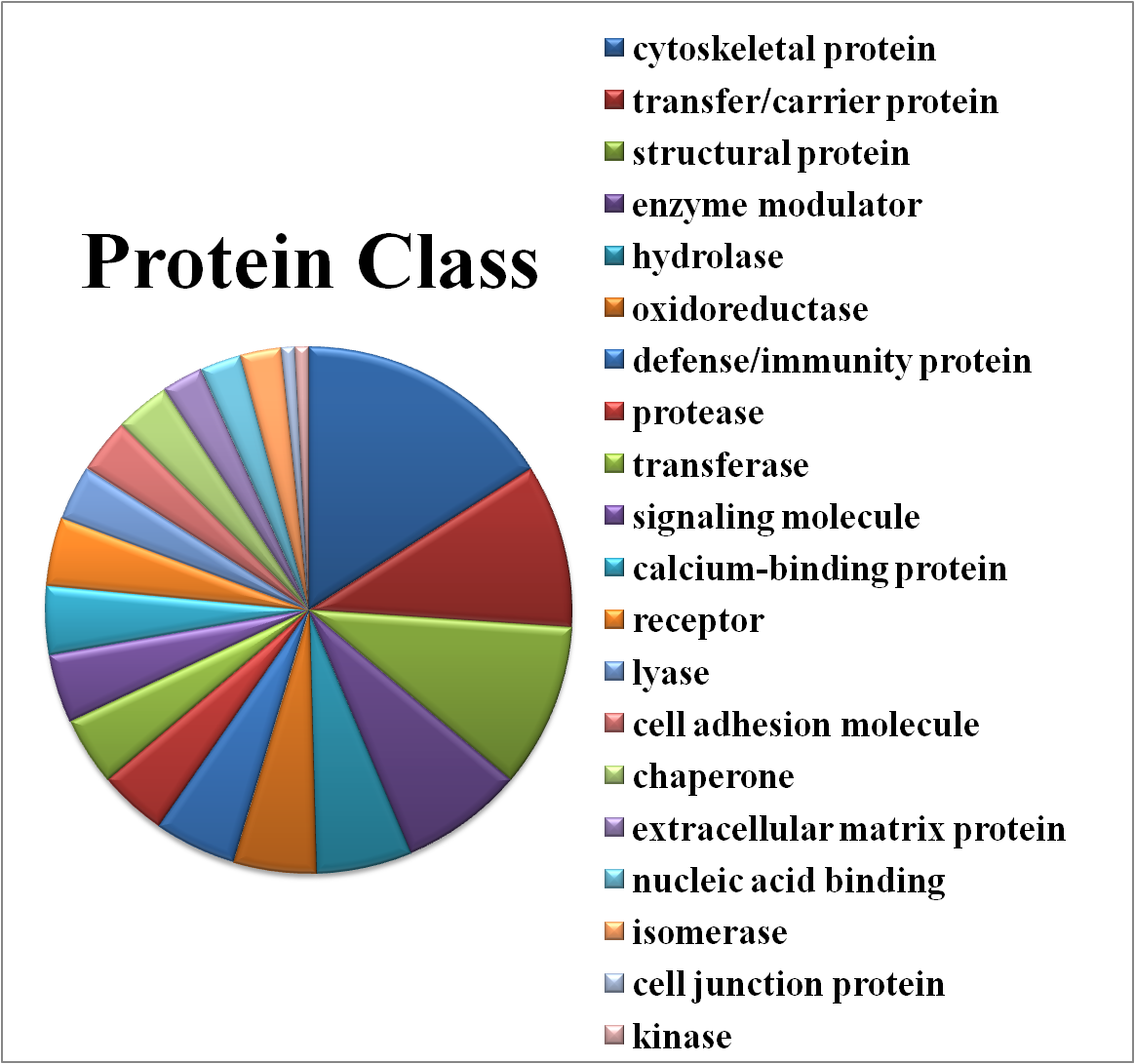 |

**Figure S2.** Gene Ontology analysis of SEVs’ proteins from lung cancer patients

| (A) | 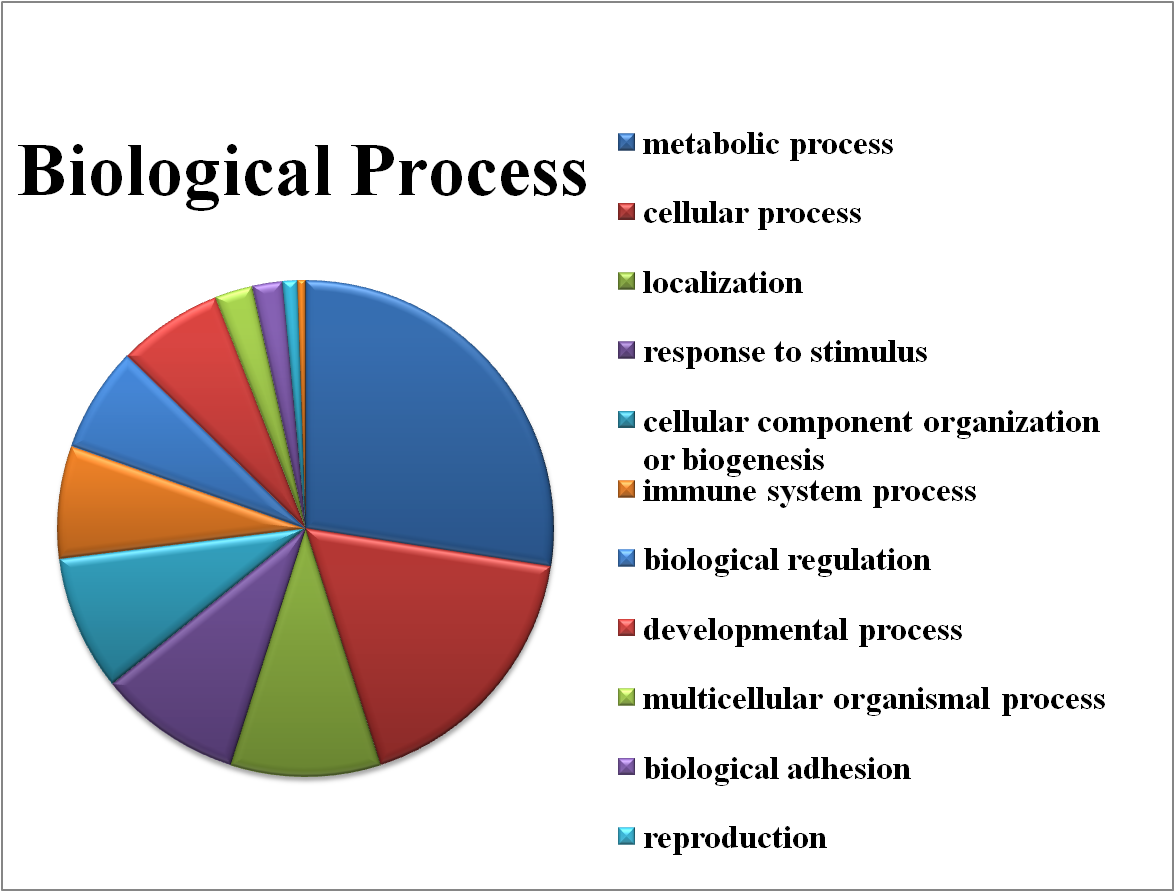 |
| --- | --- |
| (B) | 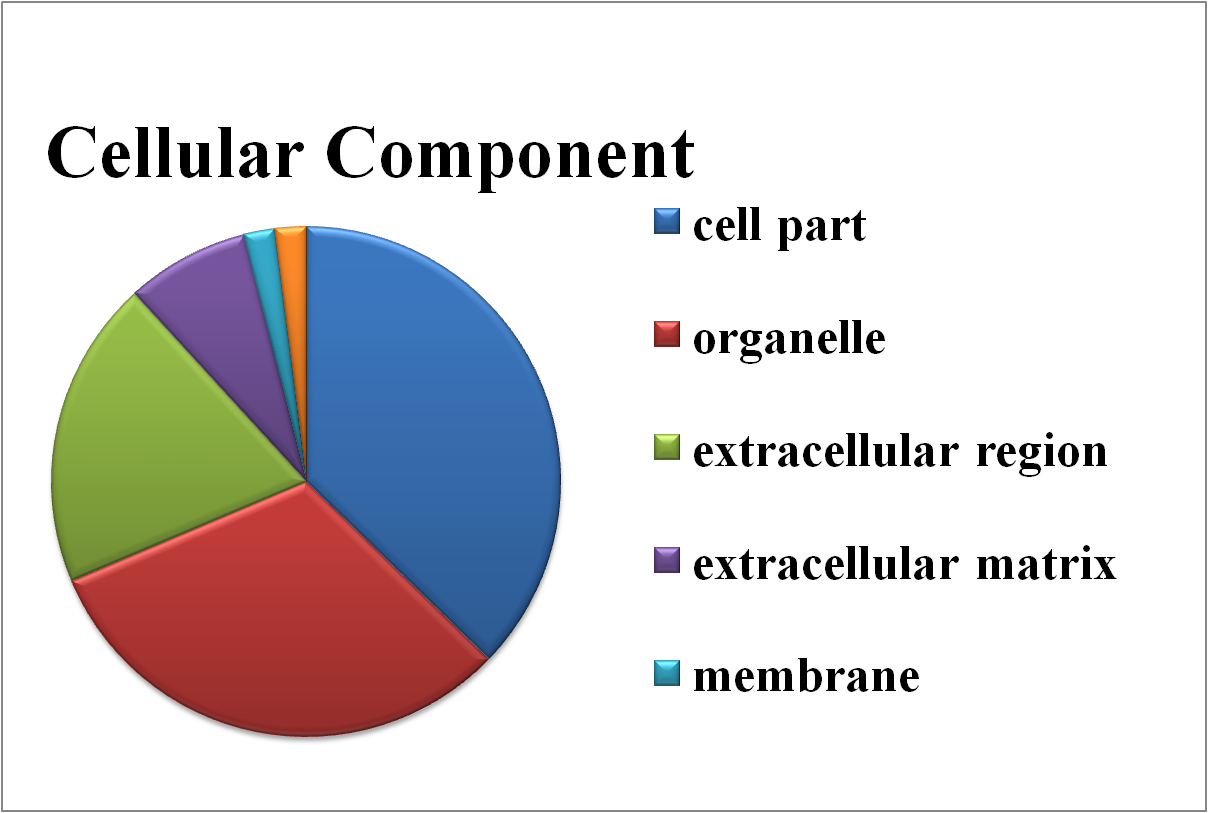 |
| (C) | 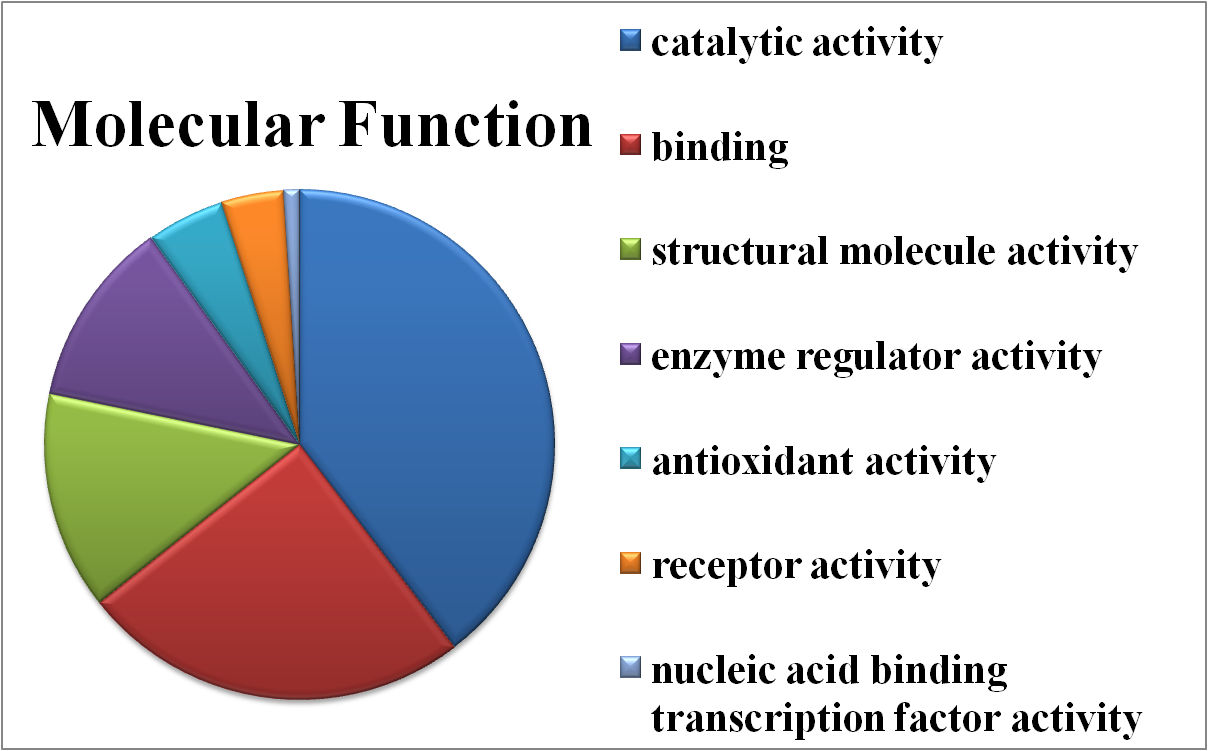 |
| D | 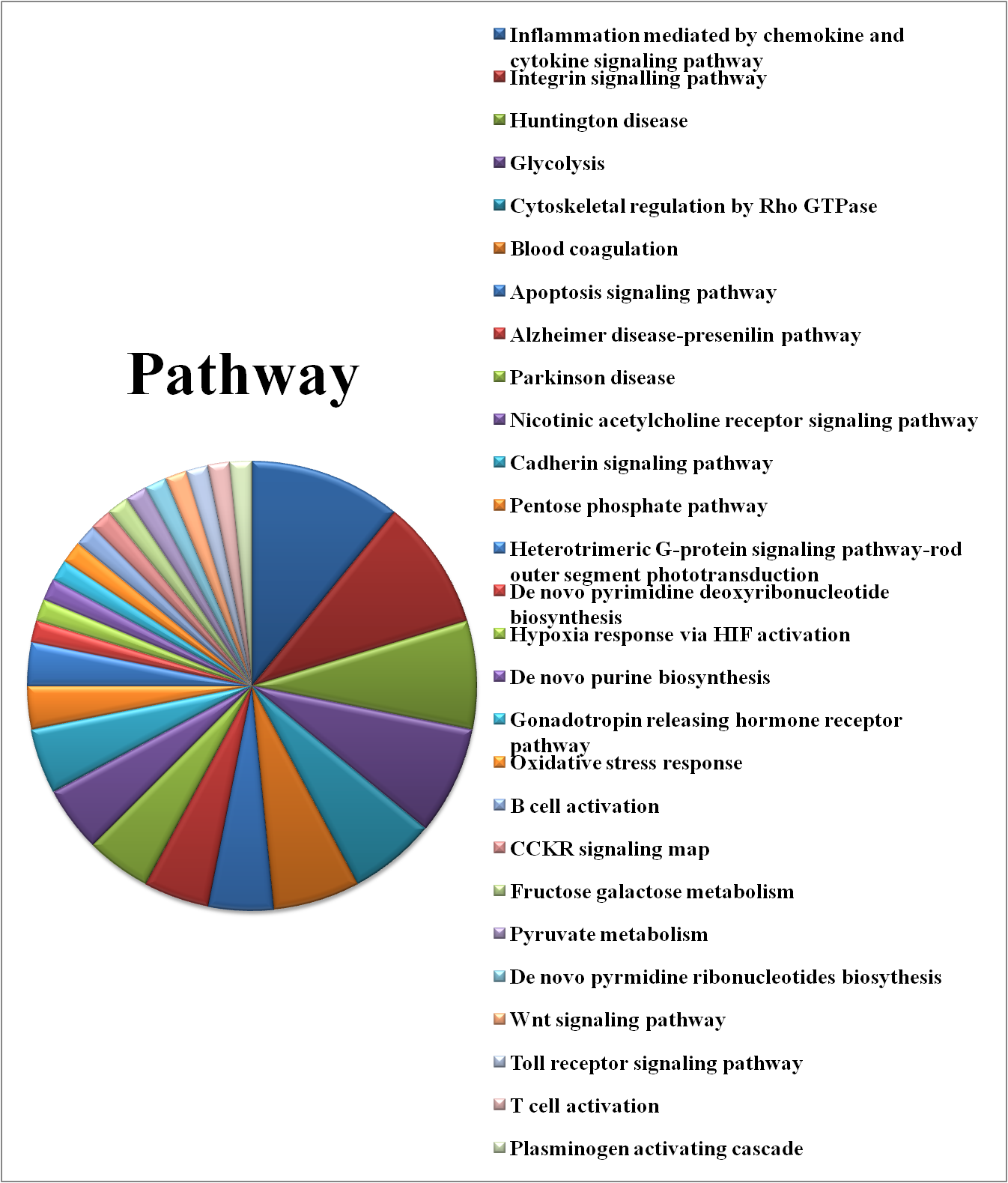 |
| E | 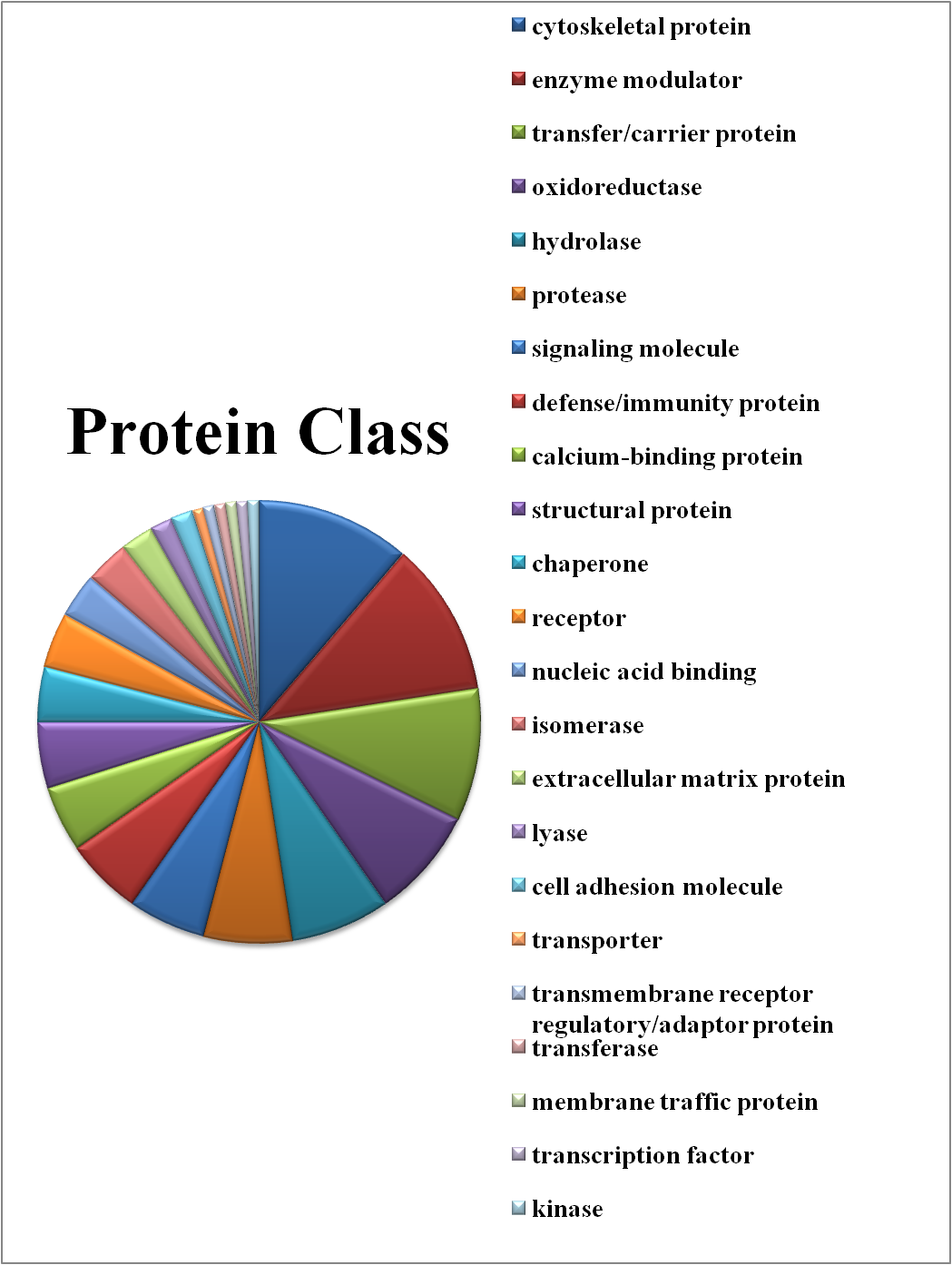 |

**Table S1. SEVs proteins that have been consistently identified from healthy donor primordial saliva by using conventional method in triplicate experiments.**

| No. | Entry | Gene  name | Protein name | Mascot score | # unique peptide | Mass (kDa) |
| --- | --- | --- | --- | --- | --- | --- |
| 1 | P01036 | CYTS | Cystatin-S | 1377 | 7 | 16.2 |
| 2 | P01591 | IGJ | Immunoglobulin J chain | 313 | 3 | 18 |
| 3 | P01620 | KV302 | Ig kappa chain V-III region SIE | 93 | 4 | 11.7 |
| 4 | P01833 | PIGR | Polymeric immunoglobulin receptor | 2707 | 2 | 83.2 |
| 5 | P01834 | IGKC | Ig kappa chain C region | 2394 | 6 | 11.6 |
| 6 | P01871 | IGHM | Ig mu chain C region | 1227 | 3 | 49.3 |
| 7 | P01876 | IGHA1 | Ig alpha-1 chain C region | 7385 | 12 | 37.6 |
| 8 | P01877 | IGHA2 | Ig alpha-2 chain C region | 1811 | 2 | 36.5 |
| 9 | P02538 | K2C6A | Keratin, type II cytoskeletal 6A | 1034 | 9 | 60 |
| 10 | P02788 | TRFL | Lactotransferrin | 2141 | 7 | 78.1 |
| 11 | P04083 | ANXA1 | Annexin A1 | 866 | 2 | 38.7 |
| 12 | P04220 | MUCB | Ig mu heavy chain disease protein | 499 | 4 | 43 |
| 13 | P04264 | K2C1 | Keratin, type II cytoskeletal 1 | 6012 | 6 | 66 |
| 14 | P04406 | G3P | Glyceraldehyde-3-phosphate dehydrogenase | 1371 | 9 | 36 |
| 15 | P04745 | AMY1 | Alpha-amylase 1 | 10222 | 14 | 57.7 |
| 16 | P04746 | AMYP | Pancreatic alpha-amylase | 6802 | 6 | 57.7 |
| 17 | P05164 | PERM | Myeloperoxidase | 309 | 2 | 83.8 |
| 18 | P07737 | PROF1 | Profilin-1 | 820 | 2 | 15 |
| 19 | P09228 | CYTT | Cystatin-SA | 962 | 2 | 16.4 |
| 20 | P0CG38 | POTEI | P | 253 | 2 | 121.2 |
| 21 | P12273 | PIP | Prolactin-inducible protein | 3031 | 2 | 16.5 |
| 22 | P13645 | K1C10 | Keratin, type I cytoskeletal 10 | 1608 | 7 | 58.8 |
| 23 | P13647 | K2C5 | Keratin, type II cytoskeletal 5 | 1617 | 4 | 62.3 |
| 24 | P14618 | KPYM | Pyruvate kinase PKM | 457 | 2 | 57.9 |
| 25 | P19012 | K1C15 | Keratin, type I cytoskeletal 15 | 164 | 2 | 49.2 |
| 26 | P19961 | AMY2B | Alpha-amylase 2B | 7951 | 13 | 57.7 |
| 27 | P22079 | PERL | Lactoperoxidase | 748 | 2 | 80.2 |
| 28 | P23280 | CAH6 | Carbonic anhydrase 6 | 6054 | 8 | 35.3 |
| 29 | P24158 | PRTN3 | Myeloblastin | 645 | 2 | 27.8 |
| 30 | P25311 | ZA2G | Zinc-alpha-2-glycoprotein | 336 | 5 | 34.2 |
| 31 | P27487 | DPP4 | Dipeptidyl peptidase 4 | 145 | 2 | 88.2 |
| 32 | P28325 | CYTD | Cystatin-D | 105 | 2 | 16 |
| 33 | P30044 | PRDX5 | Peroxiredoxin-5, mitochondrial | 109 | 2 | 22 |
| 34 | P31025 | LCN1 | Lipocalin-1 | 1130 | 2 | 19.2 |
| 35 | P35527 | K1C9 | Keratin, type I cytoskeletal 9 | 1208 | 2 | 62 |
| 36 | P35908 | K22E | Keratin, type II cytoskeletal 2 epidermal | 1737 | 9 | 65.4 |
| 37 | P54108 | CRIS3 | Cysteine-rich secretory protein 3 | 218 | 2 | 27.6 |
| 38 | P60709 | ACTB | Actin, cytoplasmic 1 | 2106 | 7 | 41.7 |
| 39 | P61626 | LYSC | Lysozyme C | 1063 | 2 | 16.5 |
| 40 | P62158 | CALM | Calmodulin | 43 | 2 | 16.8 |
| 41 | P62736 | ACTA | Actin, aortic smooth muscle | 916 | 3 | 42 |
| 42 | P68104 | EF1A1 | Elongation factor 1-alpha 1 | 149 | 2 | 50.1 |
| 43 | P81605 | DCD | Dermcidin | 309 | 7 | 11.2 |
| 44 | Q96DA0 | ZG16B | Zymogen granule protein 16 homolog B | 7042 | 9 | 22.7 |
| 45 | Q96DR5 | BPIA2 | BPI fold-containing family A member 2 | 3473 | 3 | 27 |
| 46 | Q9UGM3 | DMBT1 | Deleted in malignant brain tumors 1 protein | 2524 | 7 | 260.7 |
| 47 | P05107 | ITGB2 | Integrin beta-2 | 2016 | 5 | 84.7 |
| 48 | O75044 | SRGP2 | SLIT-R | 125 | 2 | 16 |
| 49 | P63104 | 1433Z | 14-3-3 protein zeta/delta | 885 | 2 | 40 |
| 50 | P31946 | 1433B | 14-3-3 protein beta/alpha | 734 | 2 | 22.1 |
| 51 | P59998 | ARPC4 | Actin-related protein 2/3 complex subunit 4 | 272 | 2 | 19.6 |
| 52 | P18135 | KV312 | Ig kappa chain V-III region HAH | 57 | 2 | 14.1 |
| 53 | P02768 | ALBU | Serum albumin | 4348 | 6 | 69.3 |
| 54 | Q7Z794 | K2C1B | Keratin, type II cytoskeletal 1b | 429 | 5 | 61.9 |
| 55 | Q6S8J3 | POTEE | POTE ankyrin domain family member E | 1068 | 2 | 121.3 |
| 56 | Q9BYX7 | ACTBM | Putative beta-actin-like protein 3 | 487 | 2 | 76.6 |

**Table S2. SEVs proteins that have been consistently identified from healthy donor primordial saliva by using ACCF system in triplicate experiments.**

| No. | Entry | Gene name | Protein name | Mascot score | # unique peptide | Mass (kDa) |
| --- | --- | --- | --- | --- | --- | --- |
| 1 | O75044 | SRGP2 | SLIT-R | 72 | 2 | 16 |
| 2 | P01033 | TIMP1 | Metalloproteinase inhibitor 1 | 79 | 2 | 23.1 |
| 3 | P01036 | CYTS | Cystatin-S | 1377 | 7 | 16.2 |
| 4 | P01833 | PIGR | Polymeric immunoglobulin receptor | 2707 | 2 | 83.2 |
| 5 | P01857 | IGHG1 | Ig gamma-1 chain C region | 509 | 6 | 36.1 |
| 6 | P01859 | IGHG2 | Ig gamma-2 chain C region | 357 | 4 | 35.9 |
| 7 | P01861 | IGHG4 | Ig gamma-4 chain C region | 288 | 2 | 35.9 |
| 8 | P01871 | IGHM | Ig mu chain C region | 926 | 3 | 49.8 |
| 9 | P01876 | IGHA1 | Ig alpha-1 chain C region | 7385 | 12 | 37.6 |
| 10 | P01877 | IGHA2 | Ig alpha-2 chain C region | 1811 | 2 | 36.5 |
| 11 | P02042 | HBD | Hemoglobin subunit delta | 146 | 4 | 16 |
| 12 | P02533 | K1C14 | Keratin, type I cytoskeletal 14 | 404 | 2 | 51.5 |
| 13 | P02538 | K2C6A | Keratin, type II cytoskeletal 6A | 1034 | 9 | 60 |
| 14 | P02647 | APOA1 | Apolipoprotein A-I | 644 | 4 | 30.7 |
| 15 | P02675 | FIBB | Fibrinogen beta chain | 59 | 2 | 55.9 |
| 16 | P02768 | ALBU | Serum albumin | 4348 | 6 | 69.3 |
| 17 | P02787 | TRFE | Serotransferrin | 78 | 2 | 77 |
| 18 | P02788 | TRFL | Lactotransferrin | 2141 | 7 | 78.1 |
| 19 | P04075 | ALDOA | Fructose-bisphosphate aldolase A | 510 | 4 | 65.3 |
| 20 | P04080 | CYTB | Cystatin-B | 482 | 3 | 11.1 |
| 21 | P04220 | MUCB | Ig mu heavy chain disease protein | 499 | 4 | 43 |
| 22 | P04264 | K2C1 | Keratin, type II cytoskeletal 1 | 6012 | 6 | 66 |
| 23 | P05107 | ITGB2 | Integrin beta-2 | 2365 | 5 | 84.7 |
| 24 | P05109 | S10A8 | Protein S100-A8 | 422 | 2 | 10.8 |
| 25 | P06702 | S10A9 | Protein S100-A9 | 275 | 2 | 13.2 |
| 26 | P06733 | ENOA | Alpha-enolase | 434 | 2 | 61.8 |
| 27 | P06737 | PYGL | Glycogen phosphorylase, liver form | 356 | 6 | 13.2 |
| 28 | P06870 | KLK1 | Kallikrein-1 | 98 | 4 | 28.8 |
| 29 | P07205 | PGK2 | Phosphoglycerate kinase 2 | 49 | 2 | 44.7 |
| 30 | P07602 | SAP | Prosaposin | 196 | 2 | 65.8 |
| 31 | P07737 | PROF1 | Profilin-1 | 820 | 2 | 15 |
| 32 | P08107 | HSP71 | Heat shock 70 kDa protein 1A/1B | 212 | 2 | 19.6 |
| 33 | P09228 | CYTT | Cystatin-SA | 962 | 2 | 16.4 |
| 34 | P0CG05 | LAC2 | Ig lambda-2 chain C regions | 825 | 3 | 11.2 |
| 35 | P10599 | THIO | Thioredoxin | 96 | 2 | 11.7 |
| 36 | P10909 | CLUS | Clusterin | 153 | 2 | 16.2 |
| 37 | P11216 | PYGB | Glycogen phosphorylase, brain form | 77 | 2 | 15.2 |
| 38 | P12273 | PIP | Prolactin-inducible protein | 3031 | 2 | 16.5 |
| 39 | P13645 | K1C10 | Keratin, type I cytoskeletal 10 | 1608 | 7 | 58.8 |
| 40 | P13647 | K2C5 | Keratin, type II cytoskeletal 5 | 1617 | 4 | 62.3 |
| 41 | P13796 | PLSL | Plastin-2 | 79 | 2 | 70.2 |
| 42 | P13929 | ENO3 | Beta-enolase | 265 | 2 | 46.9 |
| 43 | P14136 | GFAP | Glial fibrillary acidic protein | 356 | 2 | 49.8 |
| 44 | P14618 | KPYM | Pyruvate kinase PKM | 2094 | 7 | 53.5 |
| 45 | P19012 | K1C15 | Keratin, type I cytoskeletal 15 | 164 | 2 | 49.2 |
| 46 | P22079 | PERL | Lactoperoxidase | 748 | 2 | 80.2 |
| 47 | P22392 | NDKB | Nucleoside diphosphate kinase B | 78 | 2 | 15 |
| 48 | P23280 | CAH6 | Carbonic anhydrase 6 | 6054 | 8 | 35.3 |
| 49 | P23528 | COF1 | Cofilin-1 | 312 | 2 | 18.5 |
| 50 | P24158 | PRTN3 | Myeloblastin | 645 | 2 | 27.8 |
| 51 | P25311 | ZA2G | Zinc-alpha-2-glycoprotein | 336 | 5 | 34.2 |
| 52 | P28325 | CYTD | Cystatin-D | 105 | 2 | 16 |
| 53 | P29401 | TKT | Transketolase | 148 | 3 | 11.7 |
| 54 | P30041 | PRDX6 | Peroxiredoxin-6 | 43 | 2 | 25 |
| 55 | P30044 | PRDX5 | Peroxiredoxin-5, mitochondrial | 109 | 2 | 22 |
| 56 | P30613 | KPYR | Pyruvate kinase PKLR | 75 | 2 | 61.8 |
| 57 | P31025 | LCN1 | Lipocalin-1 | 1130 | 2 | 19.2 |
| 58 | P31146 | COR1A | Coronin-1A | 77 | 2 | 51 |
| 59 | P31946 | 1433B | 14-3-3 protein beta/alpha | 734 | 2 | 22.1 |
| 60 | P32926 | DSG3 | Desmoglein-3 | 394 | 3 | 113.7 |
| 61 | P34931 | HS71L | Heat shock 70 kDa protein 1-like | 144 | 3 | 53.1 |
| 62 | P35527 | K1C9 | Keratin, type I cytoskeletal 9 | 1208 | 2 | 62 |
| 63 | P35908 | K22E | Keratin, type II cytoskeletal 2 epidermal | 1737 | 9 | 65.4 |
| 64 | P43490 | NAMPT | Nicotinamide phosphoribosyltransferase | 102 | 2 | 11.2 |
| 65 | P52209 | 6PGD | 6-phosphogluconate dehydrogenase, decarboxylating | 491 | 2 | 70.3 |
| 66 | P60709 | ACTB | Actin, cytoplasmic 1 | 2106 | 7 | 41.7 |
| 67 | P61626 | LYSC | Lysozyme C | 1063 | 2 | 16.5 |
| 68 | P62158 | CALM | Calmodulin | 43 | 2 | 16.8 |
| 69 | P62736 | ACTA | Actin, aortic smooth muscle | 916 | 3 | 42 |
| 70 | P62937 | PPIA | Peptidyl-prolyl cis-trans isomerase A | 129 | 2 | 10.8 |
| 71 | P63104 | 1433Z | 14-3-3 protein zeta/delta | 885 | 2 | 40 |
| 72 | P68104 | EF1A1 | Elongation factor 1-alpha 1 | 149 | 2 | 50.1 |
| 73 | P68871 | HBB | Hemoglobin subunit beta | 496 | 4 | 15.9 |
| 74 | P69905 | HBA | Hemoglobin subunit alpha | 307 | 2 | 15.2 |
| 75 | P80303 | NUCB2 | Nucleobindin-2 | 467 | 4 | 55.9 |
| 76 | P81605 | DCD | Dermcidin | 309 | 7 | 11.2 |
| 77 | P98088 | MUC5A | Mucin-5AC | 104 | 4 | 585.5 |
| 78 | Q01546 | K22O | Keratin, type II cytoskeletal 2 oral | 357 | 3 | 65.8 |
| 79 | Q05639 | EF1A2 | Elongation factor 1-alpha 2 | 113 | 2 | 50.4 |
| 80 | Q08188 | TGM3 | Protein-glutamine gamma-glutamyltransferase E | 82 | 2 | 76.6 |
| 81 | Q13394 | MAB21L1 | Protein mab-21-like 1 | 567 | 2 | 40.9 |
| 82 | Q562R1 | ACTBL | Beta-actin-like protein 2 | 501 | 4 | 42 |
| 83 | Q5VSP4 | LC1L1 | Putative lipocalin 1-like protein 1 | 519 | 3 | 17.9 |
| 84 | Q5XKE5 | K2C79 | Keratin, type II cytoskeletal 79 | 1469 | 2 | 57.8 |
| 85 | Q6S8J3 | POTEE | P | 1068 | 2 | 121.3 |
| 86 | Q7Z794 | K2C1B | Keratin, type II cytoskeletal 1b | 429 | 5 | 61.9 |
| 87 | Q8N4F0 | BPIB2 | BPI fold-containing family B member 2 | 566 | 4 | 49.1 |
| 88 | Q8TDL5 | BPIB1 | BPI fold-containing family B member 1 | 1053 | 7 | 52.4 |
| 89 | Q96DA0 | ZG16B | Zymogen granule protein 16 homolog B | 7042 | 9 | 22.7 |
| 90 | Q96DR5 | BPIA2 | BPI fold-containing family A member 2 | 3473 | 3 | 27 |
| 91 | Q9BYX7 | ACTBM | Putative beta-actin-like protein 3 | 336 | 2 | 42 |
| 92 | Q9H1J1 | REN3A | Regulator of nonsense transcripts 3A | 68 | 2 | 54.6 |
| 93 | Q9HC84 | MUC5B | Mucin-5B | 29362 | 18 | 596.3 |
| 94 | Q9NP55 | BPIA1 | BPI fold-containing family A member 1 | 466 | 5 | 26.7 |
| 95 | Q9UGM3 | DMBT1 | Deleted in malignant brain tumors 1 protein | 2524 | 7 | 260.7 |

**Table S3. SEVs proteins that have been consistently identified from lung cancer patients’ primordial saliva by using ACCF system in triplicate experiments.**

| No | Entry | Entry name | protein describe | Score | unique peptide | Mass(kDa) |
| --- | --- | --- | --- | --- | --- | --- |
| 1 | O43490 | PROM1 | Prominin-1 | 220 | 4 | 98.3 |
| 2 | O75556 | SG2A1 | Mammaglobin-B | 148 | 6 | 11.1 |
| 3 | P00338 | LDHA | L-lactate dehydrogenase A chain | 115 | 3 | 36.9 |
| 4 | P00558 | PGK1 | Phosphoglycerate kinase 1 | 217 | 4 | 44.9 |
| 5 | P00738 | HPT | Haptoglobin | 75 | 2 | 45.8 |
| 6 | P01009 | A1AT | Putative lipocalin 1-like protein 1 | 519 | 4 | 18.0 |
| 7 | P01011 | AACT | Alpha-1-antichymotrypsin | 82 | 2 | 47.7 |
| 8 | P01023 | A2MG | Alpha-2-macroglobulin | 150 | 2 | 164.6 |
| 9 | P01024 | CO3 | Ig gamma-1 chain C region | 365 | 6 | 36.5 |
| 10 | P01033 | TIMP1 | Metalloproteinase inhibitor 1 | 68 | 2 | 23.8 |
| 11 | P05107 | ITGB2 | Integrin beta-2 | 2016 | 5 | 84.7 |
| 12 | P01593 | KV101 | Ig kappa chain V-I region AG | 160 | 2 | 12.0 |
| 13 | P01781 | HV320 | Ig heavy chain V-III region GAL | 75 | 2 | 12.8 |
| 14 | P01833 | PIGR | Polymeric immunoglobulin receptor | 922 | 9 | 84.4 |
| 15 | P01857 | IGHG1 | Carbonic anhydrase 6 | 407 | 4 | 35.4 |
| 16 | P01871 | IGHM | Ig mu chain C region | 115 | 3 | 49.9 |
| 17 | P01876 | IGHA1 | Ig alpha-1 chain C region | 112 | 2 | 38.4 |
| 18 | P01877 | IGHA2 | Hemoglobin subunit alpha | 441 | 5 | 15.3 |
| 19 | P02533 | K1C14 | Keratin, type I cytoskeletal 14 | 124 | 2 | 51.8 |
| 20 | P02675 | FIBB | Lactoperoxidase | 270 | 3 | 81.1 |
| 21 | P02763 | A1AG1 | Profilin-1 | 310 | 2 | 15.2 |
| 22 | P02768 | ALBU | Serum albumin | 1148 | 14 | 71.3 |
| 23 | P02787 | TRFE | Cystatin-SA | 540 | 2 | 16.7 |
| 24 | P02788 | TRFL | Lactotransferrin | 1967 | 12 | 80.0 |
| 25 | P02790 | HEMO | Hemopexin | 82 | 2 | 52.3 |
| 26 | P04040 | CATA | Catalase | 92 | 2 | 59.9 |
| 27 | P04075 | ALDOA | Fructose-bisphosphate aldolase A | 88 | 2 | 39.8 |
| 28 | P04080 | CYTB | Glyceraldehyde-3-phosphate dehydrogenase | 245 | 2 | 36.2 |
| 29 | P04083 | ANXA1 | Ig alpha-2 chain C region | 435 | 3 | 37.3 |
| 30 | P04264 | K2C1 | Keratin, type II cytoskeletal 1 | 805 | 8 | 66.1 |
| 31 | P04406 | G3P | Thioredoxin | 250 | 4 | 12.0 |
| 32 | Q8WTW4 | NPRL2 | Nitrogen permease regulator 2-like protein | 100 | 2 | 43.6 |
| 33 | P05109 | S10A8 | Protein S100-A9 | 661 | 2 | 13.2 |
| 34 | P05164 | PERM | Myeloperoxidase | 97 | 2 | 84.7 |
| 35 | P06733 | ENOA | Alpha-enolase | 141 | 2 | 47.4 |
| 36 | P06753 | TPM3 | Tropomyosin alpha-3 chain | 93 | 2 | 32.9 |
| 37 | P06870 | KLK1 | Integrin alpha-M | 300 | 2 | 128.4 |
| 38 | P07237 | PDIA1 | Protein disulfide-isomerase | 121 | 3 | 57.4 |
| 39 | P07339 | CATD | Cathepsin D | 90 | 2 | 45.0 |
| 40 | P07355 | ANXA2 | Annexin A2 | 192 | 2 | 38.8 |
| 41 | P07602 | SAP | Prosaposin | 157 | 3 | 59.8 |
| 42 | P07737 | PROF1 | Alpha-actinin-4 | 316 | 4 | 105.2 |
| 43 | P15311 | EZR | Ezrin | 43 | 2 | 69.5 |
| 44 | P08133 | ANXA6 | Annexin A6 | 159 | 2 | 76.1 |
| 45 | P08758 | ANXA5 | Annexin A5 | 68 | 2 | 35.9 |
| 46 | P09228 | CYTT | Putative beta-actin-like protein 3 | 543 | 2 | 42.3 |
| 47 | P09960 | LKHA4 | Leukotriene A-4 hydrolase | 227 | 2 | 69.8 |
| 48 | P0DMV8 | Q5JQI4 | Cystatin-B | 244 | 3 | 11.1 |
| 49 | P0DMV9 | Q5SP17 | Heat shock 70 kDa protein 1A/1B | 237 | 2 | 70.2 |
| 50 | P10599 | THIO | Cystatin-S | 254 | 2 | 16.4 |
| 51 | P11215 | ITAM | Alpha-1-acid glycoprotein 1 | 301 | 3 | 23.7 |
| 52 | P12273 | PIP | Prolactin-inducible protein | 40 | 2 | 16.8 |
| 53 | P12429 | ANXA3 | Hemoglobin subunit delta | 460 | 6 | 16.1 |
| 54 | P12814 | ACTN1 | Fibrinogen beta chain | 269 | 5 | 56.5 |
| 55 | P13645 | K1C10 | Keratin, type I cytoskeletal 10 | 161 | 2 | 59.0 |
| 56 | P13688 | CEAM1 | Carcinoembryonic antigen-related cell adhesion molecule 1 | 128 | 2 | 57.9 |
| 57 | P13796 | PLSL | Plastin-2 | 89 | 2 | 70.8 |
| 58 | P13929 | ENOB | Alpha-actinin-1 | 266 | 2 | 103.5 |
| 59 | P14174 | MIF | Macrophage migration inhibitory factor | 86 | 2 | 12.6 |
| 60 | P14618 | KPYM | Pyruvate kinase PKM | 191 | 5 | 58.4 |
| 61 | P48741 | ASPA7 | Putative heat shock 70 kDa protein 7 | 43 | 2 | 40.2 |
| 62 | P15328 | FOLR1 | Folate receptor alpha | 81 | 3 | 30.7 |
| 63 | P15941 | MUC1 | Mucin-1 | 135 | 3 | 122.2 |
| 64 | P19012 | K1C15 | Keratin, type I cytoskeletal 15 | 79 | 2 | 49.4 |
| 65 | P20061 | TCO1 | Transcobalamin-1 | 219 | 4 | 20.6 |
| 66 | P21580 | TNFAIP3 | Tumor necrosis factor alpha-induced protein 3 | 69 | 2 | 89.6 |
| 67 | P22079 | PERL | Cystatin-SN | 292 | 3 | 16.6 |
| 68 | P25311 | ZA2G | Zinc-alpha-2-glycoprotein | 177 | 3 | 34.4 |
| 69 | P26038 | MOES | Moesin | 169 | 3 | 67.8 |
| 70 | P27482 | CALL3 | Calmodulin-like protein 3 | 93 | 2 | 16.9 |
| 71 | P27487 | DPP4 | Dipeptidyl peptidase 4 | 85 | 2 | 88.9 |
| 72 | P28676 | GRAN | Grancalcin | 71 | 2 | 24.2 |
| 73 | P30041 | PRDX6 | Peroxiredoxin-6 | 76 | 2 | 25.1 |
| 74 | P30044 | PRDX5 | ADP-ribosylation factor 1 | 260 | 2 | 20.7 |
| 75 | P30740 | ILEU | Leukocyte elastase inhibitor | 194 | 2 | 42.8 |
| 76 | P31025 | LCN1 | Lipocalin-1 | 832 | 7 | 19.4 |
| 77 | P34931 | HS71L | Heat shock 70 kDa protein 1-like | 103 | 3 | 70.7 |
| 78 | P35527 | K1C9 | Keratin, type I cytoskeletal 9 | 138 | 2 | 62.2 |
| 79 | P35908 | K22E | Keratin, type II cytoskeletal 2 epidermal | 120 | 2 | 65.6 |
| 80 | P37837 | TALDO | Transaldolase | 58 | 2 | 37.6 |
| 81 | P43353 | AL3B1 | Aldehyde dehydrogenase family 3 member B1 | 75 | 3 | 52.4 |
| 82 | P50995 | ANX11 | Annexin A11 | 141 | 2 | 54.6 |
| 83 | P52209 | 6PGD | 6-phosphogluconate dehydrogenase, decarboxylating | 140 | 2 | 53.6 |
| 84 | P52566 | GDIR2 | Rho GDP-dissociation inhibitor 2 | 98 | 2 | 23.0 |
| 85 | P54652 | HSP72 | Heat shock-related 70 kDa protein 2 | 147 | 2 | 70.2 |
| 86 | P60709 | ACTB | Actin, cytoplasmic 1 | 148 | 2 | 42.0 |
| 87 | P61626 | LYSC | Lysozyme C | 228 | 2 | 16.9 |
| 88 | P62158 | CALM | Calmodulin | 185 | 3 | 16.8 |
| 89 | P62736 | ACTA | Actin, aortic smooth muscle | 100 | 3 | 42.3 |
| 90 | P62805 | H4 | Histone H4 | 195 | 2 | 11.3 |
| 91 | P62937 | PPIA | Peptidyl-prolyl cis-trans isomerase A | 116 | 2 | 18.2 |
| 92 | P68871 | HBB | Hemoglobin subunit beta | 44 | 2 | 16.1 |
| 93 | P69905 | HBA | Annexin A3 | 455 | 2 | 36.5 |
| 94 | P80188 | NGAL | Neutrophil gelatinase-associated lipocalin | 150 | 2 | 22.7 |
| 95 | P84077 | ARF1 | Beta-enolase | 264 | 2 | 47.2 |
| 96 | Q01469 | FABP5 | Alpha-1-antitrypsin | 502 | 6 | 46.8 |
| 97 | Q06830 | PRDX1 | Peroxiredoxin-1 | 104 | 2 | 22.3 |
| 98 | Q13421 | MSLN | Mesothelin | 58 | 2 | 69.6 |
| 99 | Q562R1 | ACTBL | Beta-actin-like protein 2 | 80 | 2 | 42.3 |
| 100 | Q58FF8 | H90B2 | Putative heat shock protein HSP 90-beta 2 | 33 | 2 | 44.4 |
| 101 | Q58FG1 | HS904 | Putative heat shock protein HSP 90-alpha A4 | 36 | 2 | 47.7 |
| 102 | Q5SNV9 | CA167 | Uncharacterized protein C1orf167 | 125 | 2 | 164.6 |
| 103 | Q5VSP4 | LC1L1 | Serotransferrin | 529 | 2 | 79.2 |
| 104 | Q687X5 | STEA4 | Metalloreductase STEAP4 | 80 | 2 | 52.5 |
| 105 | Q6MZM9 | PRR27 | Proline-rich protein 27 | 46 | 2 | 22.8 |
| 106 | Q8N4F0 | BPIB2 | BPI fold-containing family B member 2 | 88 | 2 | 49.2 |
| 107 | P01034 | CYTC | Cystatin-C | 126 | 2 | 15.7 |
| 108 | Q8TDL5 | BPIB1 | BPI fold-containing family B member 1 | 713 | 5 | 52.5 |
| 109 | Q96A08 | H2B1A | Histone H2B type 1-A | 91 | 2 | 14.1 |
| 110 | Q96DA0 | ZG16B | Zymogen granule protein 16 homolog B | 671 | 5 | 22.7 |
| 111 | Q96QR1 | SG3A1 | Secretoglobin family 3A member 1 | 131 | 2 | 10.2 |
| 112 | Q9GZZ8 | LACRT | Extracellular glycoprotein lacritin | 120 | 2 | 14.2 |
| 113 | Q9HC84 | MUC5B | Mucin-5B | 1265 | 10 | 611.5 |

**Table S4.** IPA network analysis of candidate SEVs’ protein biomarkers for lung cancer

| Network ID | Molecules in Network | Score | Focus Molecules | Top Diseases and Functions |
| --- | --- | --- | --- | --- |
| 1 | Actin,**ACTN1,ANXA1,ANXA2,ANXA5,ARHGDIB,C3,CAT,CEACAM1,EZR,**F Actin,Fibrinogen,**GAPDH**,HDL,Histone h3,**HP,HPX,**Hsp70,Hsp90,**HSPA2,HSPA1A/HSPA1B,ITGAM,**LDL,**MIF,MPO,MSN,MUC1,ORM1**,P glycoprotein**,PGK1,PRDX1**,Rho gdi,**SERPINA1,TNFAIP3,TPM3** | 59 | 25 | Cellular Movement, Immune Cell Trafficking, Inflammatory Response |
| 2 | **ANXA3,ANXA6,CALML3**,CAMK2N1,CPOX,CTIF,**DPP4**,ELAVL1,EZH2,**FABP5**,FUT4,**GCA**,GLIPR1,ISG15**,LACRT,LTA4H,**MARVELD2,MBNL2,MPV17L,**P4HB**,PANK1,**SCGB2A1**,SELT,**SERPINA3,SERPINB1**,SLC19A2,SLC39A10,STAT3,**TALDO1**,TMED7,TMEM127,TP53,UBL3,WWOX,ZNF668 | 25 | 13 | Cancer, Cell Cycle, Organismal Injury and Abnormalities |
| 3 | ACYP1,AIFM3,**ALDH3B1**,ALDH8A1,**ANXA11**,APP,**ARF1**,CASZ1,**CST3,CTSD**,CUL5,DYNC1H1,FETUB,**FOLR1**,FOS,GKN1,**HIST1H2BA**,HOXA9,HSP90AA1,HTT,**LDHA,MSLN**,MTURN,NAT6,NME8,**NPRL2**,**PROM1**,PUS7L,RNF151,**SCGB3A1**,SUN5,TBCC,**TCN1**,UBC,ZNF22 | 25 | 13 | Neurological Disease, Cell Morphology, Hematological System Development and Function |
| 4 | **A2M**,B2M,CCT5,CFLAR,CSF3,CXCL8,DNAJB1,ENO2,GTF2B,HLA-A,HMGB1,HMOX1,HSF1,HSF2,**HSPA7**,IL2,IL4,IL6,IL1B,**LCN2**,MCL1,MICA,MMP9,NFkB (complex),NFKBIZ,PTGES3,RAD23A,RPL22,SNRPD3,STAT,**STEAP4**,TERT,TNFSF11,TTR,TUBA1B | 6 | 4 | Cell-To-Cell Signaling and Interaction, Hematological System Development and Function, Immune Cell Trafficking |

Notes: Protein names in **bold** were identified uniquely in the SEVs of lung cancer patients.
